# Metal–Organic Framework‐Surface‐Enhanced Infrared Absorption Platform Enables Simultaneous On‐Chip Sensing of Greenhouse Gases

**DOI:** 10.1002/advs.202001173

**Published:** 2020-09-18

**Authors:** Hong Zhou, Xindan Hui, Dongxiao Li, Donglin Hu, Xin Chen, Xianming He, Lingxiao Gao, He Huang, Chengkuo Lee, Xiaojing Mu

**Affiliations:** ^1^ Key Laboratory of Optoelectronic Technology & Systems Ministry of Education, and International R & D Center of Micro‐Nano Systems and New Materials Technology Chongqing University Chongqing 400044 P. R. China; ^2^ Suzhou Institute of Nano‐Tech and Nano‐Bionics Chinese Academy of Sciences Suzhou 215123 P. R. China; ^3^ Department of Electrical and Computer Engineering National University of Singapore Singapore 117583 Singapore

**Keywords:** greenhouse gases, metal–organic frameworks, metamaterial absorbers, multigas sensors, surface‐enhanced infrared absorption

## Abstract

Simultaneous on‐chip sensing of multiple greenhouse gases in a complex gas environment is highly desirable in industry, agriculture, and meteorology, but remains challenging due to their ultralow concentrations and mutual interference. Porous microstructure and extremely high surface areas in metal–organic frameworks (MOFs) provide both excellent adsorption selectivity and high gases affinity for multigas sensing. Herein, it is described that integrating MOFs into a multiresonant surface‐enhanced infrared absorption (SEIRA) platform can overcome the shortcomings of poor selectivity in multigas sensing and enable simultaneous on‐chip sensing of greenhouse gases with ultralow concentrations. The strategy leverages the near‐field intensity enhancement (over 1500‐fold) of multiresonant SEIRA technique and the outstanding gas selectivity and affinity of MOFs. It is experimentally demonstrated that the MOF–SEIRA platform achieves simultaneous on‐chip sensing of CO_2_ and CH_4_ with fast response time (<60 s), high accuracy (CO_2_: 1.1%, CH_4_: 0.4%), small footprint (100 × 100 µm^2^), and excellent linearity in wide concentration range (0–2.5 × 10^4^ ppm). Additionally, the excellent scalability to detect more gases is explored. This work opens up exciting possibilities for the implementation of all‐in‐one, real‐time, and on‐chip multigas detection as well as provides a valuable toolkit for greenhouse gas sensing applications.

## Graphical abstract

Porous metal–organic frameworks are provided with excellent gas adsorption selectivity due to their extremely high surface areas. Integrating it into the multiresonant surface‐enhanced infrared absorption platform overcomes the shortcomings of poor selectivity in gas sensing and enables simultaneous on‐chip sensing of greenhouse gases. Importantly, the strategy shows a competitive advantage in terms of response time, accuracy, footprint, and linearity.

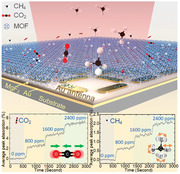

## 1 Introduction

Gas sensing is one of the most ubiquitous and significant technologies and plays a vital role in many applications in our daily life.^[^ ^1^ ^]^ In many special scenarios, there is a huge demand for the simultaneous sensing of multiple gases, such as the detection of combustible gases accompanied with the monitor of oxygen (O_2_) to prevent explosions, the gauging of multiple toxic gases in small and confined spaces (like a vehicle), real‐time monitor of O_2_ and carbon dioxide (CO_2_) in the farm, and the simultaneous sensing of multiple greenhouse gases in industry and meteorology.^[^ ^2^ ^]^ Many efforts have been invested to address such challenges, mainly including i) electrochemical methods exploiting electrochemical redox reaction^[^ ^3^ ^]^ and ii) optical methods including spectrophotometry, photoacoustic spectroscopy, tunable diode laser spectroscopy, and IR absorption spectroscopy.^[^ ^4^ ^]^ Among them, nondispersive infrared (NDIR) sensors using IR absorption spectroscopy stand out and are widely commercialized due to its fast response, no poisoning effects, and long lifespan.^[^ ^5^ ^]^ However, such device is often bulky because it requires centimeter long optical interaction length to achieve ppm detection level limit.^[^ ^6^ ^]^ Despite the introduction of metamaterial technology to NDIR to reduce its size and enhance its performance, the problems of low sensitivity, poor selectivity, and interference remain unresolved due to the inherent limitation of its basic sensing principles.^[^ ^7^ ^]^ Furthermore, in order to achieve multiple gas detection, such approach requires assembling several different gas‐sensitive detectors together as a NDIR system, i.e., a multigas meter, which results in a bulkier shell. Therefore, a new technology for enabling multiple gas detection in a miniaturized device footprint of excellent selectivity, high sensitivity, and great accuracy is still required.

The surface‐enhanced infrared absorption (SEIRA) technique can potentially address the challenge by utilizing the near‐field coupling between plasmonic resonances and vibrational modes of target molecules.^[^ ^8^ ^]^ Meanwhile, the gas‐selective‐trapping material is integrated with SEIRA platform to concentrate the sparsely dispersed gas molecules of the surrounding area.^[^ ^9^, ^10^, ^11^ ^]^ The gas‐selective‐trapping material is critical because it determines the performance of the sensor, including selectivity, response time, linearity, and sensitivity. For instance, polyethylenimine was integrated with a hybrid metamaterial absorber to achieve high sensitivity sensing of CO_2_.^[^ ^9^ ^]^ Palladium was chosen as hydrogen trapping material in a plasmonic sensing system to realize the sensing of hydrogen due to the formation of palladium‐hydride upon exposure to hydrogen.^[^ ^10^ ^]^ More recently, metal–organic framework (MOF) was reported for CO_2_ sensing due to the porous structure and selectivity.^[^ ^11^ ^]^ Although these studies demonstrated the feasibility of SEIRA integrated with functional films for miniaturized, highly sensitive, and selective gas sensing, multigas sensing with this strategy has not been well studied. The difficulties associated with the multigas detection lie in both the development of functional material with multigas selectivity and the implementation of multiresonant SEIRA platform compatible with functional material and target gases.

To address these grand challenges, we hereby develop a miniaturized multigas sensor by combining the zeolitic imidazolate framework (ZIF‐8) and the multiresonant plasmonic SEIRA platform. Its multigas detection capability is demonstrated by the simultaneous on‐chip sensing of greenhouse gases, mainly CO_2_ and CH_4_, which are accounted for the majority of global warming.^[^ ^12^ ^]^ The successful implementation of simultaneous multigas detection comes from two effects: the multiple selectivity of ZIF‐8 and the near‐field intensity enhancement of the multiresonant SEIRA. On the one hand, the porous ZIF‐8 shows excellent gas‐selective‐trapping features for both CO_2_ and CH_4_. This interesting feature is determined by both the pore aperture and cavity diameter of ZIF‐8 and the kinetic diameter of CO_2_ and CH_4_.^[^ ^13^ ^]^ Since the kinetic diameter of CO_2_ and CH_4_ are 3.3 and 3.8 Å, respectively, CH_4_ is only adsorbed in the cavity (diameter: 11.6 Å), while CO_2_ can enter the pore aperture (diameter: 3.4 Å). It indicates that most of the CO_2_ and CH_4_ are captured in different areas of the ZIF‐8. Therefore, the competition between CO_2_ and CH_4_ in ZIF‐8 is relatively small, which is a critical characteristic for the simultaneous detection of the gases. Moreover, the multiresonant SEIRA technique used in the proposed MOF–SEIRA platform provides more than 1500‐fold near‐field intensity enhancement over dual sensing bands corresponding to CO_2_ and CH_4_ concentrated in ZIF‐8. Notably, in addition to selectively eliminating the interference of nontarget gas molecules in the environment through ZIF‐8, the platform can further identify CO_2_ and CH_4_ through the vibration peaks in the mid‐IR spectrum. This dual recognition feature adds extra advantages to our MOF–SEIRA platform. Collectively, this work primarily focuses on the detailed investigation of the MOF–SEIRA platform including its optical physics and sensing performance, such as mechanism, theoretical models, steady‐state, and dynamic sensing behaviors. We believe that these findings will provide both a powerful detection tool for greenhouse gases sensing and an enabling SEIRA platform technology in simultaneous selective sensing of gases.

## 2 Results and Discussions

### 2.1 Mechanism of MOF–SEIRA Platform

**Figure 1:**
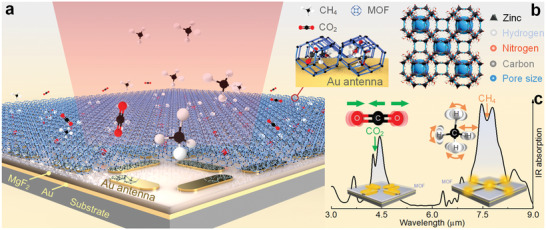
Illustration of the MOF–SEIRA platform for simultaneous sensing of CO_2_ and CH_4_ gases. a) Schematic representation of the MOF–SEIRA platform consisting of porous ZIF‐8 and metamaterial perfect absorber. Inset: CO_2_ and CH_4_ molecules are simultaneously trapped in the cavity and pore aperture of the ZIF‐8, and the MOF is attached to the surface of the antenna covered by the hotspot. b) A stick diagram showing the structure of ZIF‐8 with zinc–nitrogen tetrahedra. c) The absorber resonance position is engineered to overlap with the vibration signals of both the CO_2_ and CH_4_ absorption bands at the same time, achieving the simultaneous sensing of CO_2_ and CH_4_ gases.

The proposed MOF–SEIRA platform, illustrated in **Figure** **1**a, combines the SEIRA platform and the porous MOF for simultaneous on‐chip sensing of CO_2_ and CH_4_ at mid‐IR spectra. Here, the MOF film functions as a multigas‐selective material that selectively adsorbs gases due to its unique structural characteristics, high surface area, chemical, and thermal stability.^[^ ^13^ ^]^ In particular, ZIF‐8, a subclass of MOFs, is composed of Zn^2+^ atoms linked to imidazolate anions through nitrogen and presents a tetrahedral coordination, as shown in Figure 1b. The largest cavity in the nets of the ZIF structure is 11.6 Å, and the cavity is connected through small apertures (3.4 Å, formed by the encompassment of six‐membered ring window), as shown in Figure S1 (Supporting Information). Its pore aperture and cavity size determine the characteristics of simultaneously capturing and concentrating CH_4_ and CO_2_ molecules of the surrounding area. The above analysis of the competition between CO_2_ and CH_4_ will be experimentally demonstrated in the later section.

After the adsorption and concentration of target gases through MOF, they are coupled with the enhanced near‐field provided by the metamaterial absorber. The metamaterial absorber consists of metal‐dielectric spacer‐metal layers, i.e., gold metasurface layer, MgF_2_ dielectric spacer, and gold ground layer. Such a trilayer structure forms a Fabry–Perot cavity where multiple reflections occur to achieve absorption effects and field enhancement. Perfect absorption occurs when the impedance is matched with free space by tuning the thickness of dielectric spacer to optimize intrinsic and external loss rates.^[^ ^14^ ^]^ Such perfect absorption improves SEIRA performance, and thereby provides an enhanced near‐field coupling platform to detect the weak intrinsic signals originating from picometer‐sized gas molecules. The enhancement achieved by metamaterial absorber have proven to increase sensitivity by an order of magnitude compared with metasurface working in the transmission mode.^[^ ^15^ ^]^ In addition, the resonance frequency of the SEIRA platform is tuned by the shape and dimension of the patterned nanostructures. The nanostructures are engineered as symmetrical cross‐shaped antennas, consisting of two sets of nanorods perpendicular to each other. It provides resonance at both 4.25 and 7.66 µm, one excited at the ends and another excited at the sides, for sensing vibrational fingerprint signals derived from gas molecules, as shown in the insets of Figure 1c. Furthermore, in order to increase the strength of the near‐field coupling, the two resonance of mid‐IR absorber is artificially tailored to overlap with the vibrational modes of the CH_4_ and CO_2_ molecules, as plotted in Figure 1c. The spectral position of CO_2_ used in this work is fixed at 4.25 µm (2350 cm^−1^) induced by the asymmetrical stretch of C=O=C, and that of CH_4_ gas at 7.66 µm (1305.9 cm^−1^) originating from the *υ*_4_ vibration band of methane.^[^ ^16^ ^]^ Collectively, the sensing of target gases involves two steps: CH_4_ and CO_2_ molecules of the surrounding area diffuse and adsorb in ZIF‐8; they are then detected by utilizing resonant plasmonic and vibrational coupling in the platform.

### 2.2 Simulation, Modeling, and Proof‐of‐Concept Demonstration of MOF–SEIRA Platform

**Figure 2:**
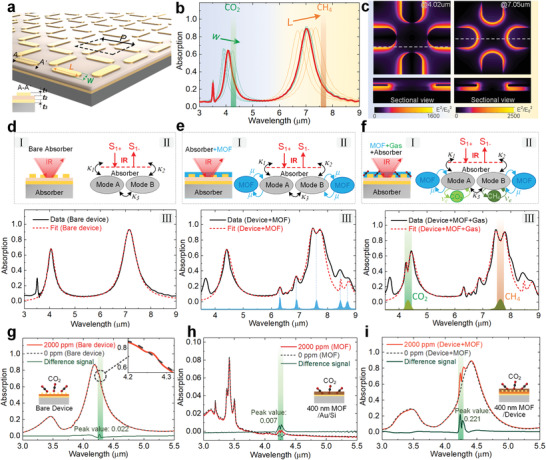
Simulation, modeling, and proof‐of‐concept demonstration of the MOF–SEIRA platform. a) Schematic of the multiresonant metamaterial absorber. Dimensions: *L* = 2.3 µm, *W* = 1 µm, *P* = 3.5 µm, *t*_1_ = 100 nmL, *t*_2_ = 200 nm, *t*_3_ = 100 nm. b) Simulated absorption spectrum of the MOF–SEIRA platform for the nominal configuration (red curve), and with varying width *W* (green curves, 0.9 ≤ *W* ≤ 1.1) and length *L* (orange curves, 2.27 ≤ *L* ≤ 2.43). c) Near‐field distribution of the MOF–SEIRA platform parallel (upper) and perpendicular (bottom) to the substrate plane at the CO_2_ (left) and CH_4_ (right) bands. d) Generalized absorber model for the metamaterial absorber. I: Schematic of the metamaterial absorber. II: Generalized absorber model. III: Simulated absorption spectra and model fit spectra. e) Extension of generalized model for the MOF–SEIRA platform. f) Corresponding model when CO_2_ and CH_4_ are loaded. *κ*_1_, *κ*_2_, *κ*_3_, *µ*, and *υ* represent coupling coefficient. g) Measured absorption spectra for bare metamaterial absorber, h) MOF thin film, and i) MOF–SEIRA platform in vacuum and 2000 ppm CO_2_. The thickness of MOF on both the absorber and the Au/Si were set to 400 nm.

The optical response of the MOF–SEIRA platform is investigated using 3D finite‐difference time‐domain (FDTD) method. **Figure** **2**a depicts the 3D structure of SEIRA platform, in which a plane wave light source is incident from the top. Since transmission is nearly zero across the entire frequency range due to the metallic ground plane which is thicker than the penetration depth of light in the IR range, the absorption *A* is calculated as 1 – reflection *R*, and the absorption characteristics of absorbers are studied by reflection spectroscopy (Figure S2, Supporting Information). The simulated absorption spectra of SEIRA platform containing two well‐designed resonances are plotted in Figure 2b. Considering that significant redshifts will occur on the resonance after integrating the gas‐selective‐trapping MOF into the SEIRA platform, the two resonances are elaborately designed to the left side of the vibration signals of CO_2_ and CH_4_ to reserve some spectral space. It is achieved by adjusting the length *L* and width *W*, as shown in Figure 2b. *L* and *W* correspond to the low and high frequencies of the absorber, respectively, and only the corresponding resonance changes when adjusting *L* and *W*, indicating that the two modes of cross‐shaped antennas are weakly coupled. This feature not only allows the resonances to be engineered to desired bands in a straightforward manner, but also minimizes the interference between the two resonances.

The enhanced near‐field in the vicinity of nanoantennas is significant in SEIRA due to its critical role in the light‐matter interaction. The electric field distribution in Figure 2c shows that resonance is excited in different areas of the nanoantennas for each spectral band corresponding to vibrational signal of gases, indicating that the sensitive areas of each gas on the platform are separated. In addition, the metamaterial absorber‐based SEIRA platform creates maximum local near‐field intensity enhancements over 1500‐fold for both sensing bands, which is about 10 times higher than that of metasurface‐based SEIRA platform working in the transmission mode (Figure S2, Supporting Information). Importantly, the near‐field access up to hundreds of nanometers from the dielectric surface with 10% of the near‐field peak intensity (Figure S3, Supporting Information), which is much deeper than the penetration depth realized by surface‐enhanced Raman spectroscopy. Such extended penetration depth is independent of the absorber geometry and provides a relatively sufficient volume for the light‐matter interaction (Figure S4, Supporting Information).

In order to better understand the mechanism of the proposed platform, generalized absorber model is established according to the temporal coupled mode theory (TCMT).^[^ ^17^ ^]^ Corresponding to the three evolutionary processes of the platform, the model is built in three steps, namely the metamaterial absorber model, the MOF–SEIRA platform model, and the gases sensing model. For the metamaterial absorber (Figure 2dI), the theoretical model consists of a dual‐mode (labeled mode A and mode B) optical absorber coupled with two ports representing the incoming (S_1+_) and outgoing (S_1−_) radiation (Figure 2dII). According to the coupling equations of TCMT, the absorption *A*_1_ can be written as (Note S4.1, Supporting Information)

(1) $$\begin{array}{c} A_{1}=1-{\left|1-\frac{\kappa_{1}{}^{2}Y_{1}+\kappa_{2}{}^{2}Y_{2}+j\kappa_{3}{\left(\kappa_{1}-\kappa_{2}\right)}^{2}}{Y_{1}Y_{2}+j\kappa_{3}\left(Y_{1}+Y_{2}\right)}\right|}^{2} \end{array}$$

where *Y_i_* = *j*(*ω* − *ω*_*i*_) + *γ*_*i*_ − *jκ*_3_ (*i* = 1, 2), and the terms involved include center frequency (*ω*_1_ for mode A and *ω*_2_ for mode B), damping rates (*γ*_1_, *γ*_2_), and coupling coefficient (*κ*_1_, *κ*_2_, *κ*_3_). An absorption spectrum containing two Lorentzian line shapes can be obtained by plotting Equation (1) in MATLAB software. The resonance parameters determining the line‐width and amplitude are extracted by fitting Equation (1) to the simulated spectrum, as shown in Figure 2dIII. The values of the parameters in Equation (1) are listed in Table S2 (Supporting Information). The coupling coefficient *κ*_3_ between mode A and mode B has little effect on the absorption spectrum when the resonant frequencies of the two modes is significantly different (Figure S5, Supporting Information).

For the MOF–SEIRA platform (Figure 2eI), the above model is extended via TCMT to include the effects of the MOF thin film. It is achieved by coupling (coefficient *µ*) a purely dissipative mode representative of the MOF absorption to the absorber model (Figure 2eII). According to the IR absorption spectrum of MOF (Figure S8, Supporting Information), there are five peaks in the spectral band of interest, i.e., 1148, 1182, 1314, 1449, and 1583 cm^−1^, as shown at the bottom of Figure 2eIII. All of them can excite dissipative mode in the band to affect the model, so the absorption *A*_2_ with dissipative MOF considered can be expressed as (Note S4.2, Supporting Information)

(2) $$\begin{array}{ccc} A_{2} & = & 1-\left|1-\frac{\kappa_{1}^{2}}{j\left(\omega -\omega_{1}\right)+\gamma_{1}}\right.\\ & & -\;{\left.\frac{\kappa_{2}^{2}}{j\left(\omega -\omega_{2}\right)+\gamma_{2}+\Gamma_{1}+\Gamma_{2}+\Gamma_{3}+\Gamma_{4}+\Gamma_{5}}\right|}^{2} \end{array}$$

where $\Gamma_{i}=\mu_{bi}^{2}/\left[j\left(\omega -\omega_{bi}\right)+\gamma_{bi}\right]\;\left(i=1,2,3,4,5\right)$, and the terms involved include absorption frequencies of MOF (labeled *ω*_b1_,…, *ω*_b5_) and corresponding damping rates (labeled *γ*_b1_,…, *γ*_b5_). Compared with absorption *A*_1_ in the metamaterial absorber model, *A*_2_ adds the term Г*_i_* representing the absorption of MOF. The spectrum extracted from the model fits well with the simulated spectrum, indicating the correctness and accuracy of the model established, as shown in Figure 2eIII.

When MOF–SEIRA platform is exposed to ambient CO_2_ and CH_4_ sorption (Figure 2fI), a gases sensing model is established by coupling (coefficient *υ*_c_, *υ*_e_) the dissipative mode representing gas absorption to the MOF–SEIRA platform model. According to the interaction between gases vibration and platform resonances, CO_2_ and CH_4_ are coupled to mode A and mode B of the MOF–SEIRA platform, respectively, as shown in Figure 2fII. According to the coupling equations of TCMT, the absorption *A*_3_ can be expressed as (Note S4.3, Supporting Information)

(3) $$\begin{array}{ccc} A_{3} & = & 1-\left|1-\frac{\kappa_{1}^{2}}{j\left(\omega -\omega_{1}\right)+\gamma_{1}+Y_{c}}\right.\\ & & -{\left.\frac{\kappa_{2}^{2}}{j\left(\omega -\omega_{2}\right)+\gamma_{2}+\Gamma_{1}+\Gamma_{2}+\Gamma_{3}+\Gamma_{4}+\Gamma_{5}+Y_{e}}\right|}^{2} \end{array}$$

where $Y_{c,e}=\upsilon_{c,e}^{2}/\left[j\left(\omega -\omega_{c,e}\right)+\lambda_{c,e}\right]$, and the added terms include absorption frequencies of CO_2_ (*ω*_c_) and CH_4_ (*ω*_e_) and corresponding damping rates (*γ*_c_, *γ*_e_). Compared with *A*_2_, *A*_3_ adds the terms *Y*_c,e_ representing the absorption of CO_2_ and CH_4_. The signal peak corresponding to gas absorption can be clearly observed in the spectrum, as shown in Figure 2fIII.

To investigate the enhancement effect of the platform, the metamaterial absorber, ZIF‐8 film and MOF–SEIRA platform were exposed to ambient CO_2_ sorption (2000 ppm) while keeping other experimental conditions consistent. Figure 2g shows the spectral response of metamaterial absorber. The signal corresponding to CO_2_ absorption was weak and even negligible in the spectrum. The difference signal was extracted by setting the spectrum measured in vacuum as a reference. A peak value of 0.022 was observed in the difference spectrum, which is due to the small amount of CO_2_ in the near field. Figure 2h exhibits the CO_2_ sensing behavior of the ZIF‐8 film on a gold substrate without any pattern. A distinct peak was observed at around 4.25 µm in the measured spectrum, while the signal intensity is still weak (less than 1.5% absorption) due to the short optical interaction length. Figure 2i is the proof‐of‐concept demonstration of CO_2_ sensing using the MOF–SEIRA platform. A highly visible signal representing CO_2_ absorption appeared in the spectrum when the platform is exposed to ambient CO_2_ from a vacuum environment. The peak value of the MOF–SEIRA platform is 30 times higher than that of the MOF film, and 10 times higher than the metamaterial absorber. This phenomenon was attributed to the much higher enhancement depth and field intensity of the MOF–SEIRA platform and could be analyzed from two aspects, namely the concentration of gas by MOF and the enhancement of near‐field by metamaterial absorber. From the perspective of the generalized model, the coupling coefficient *υ*_c,e_ between the gas vibration and the absorber resonance is enlarged due to the concentration of gas by MOF. In addition, CO_2_ molecules in MOF–SEIRA platform mainly consume IR energy in the metamaterial absorber through near‐field coupling, rather than directly absorbing IR energy as in the MOF/Au/Si configuration. Such mechanism is a key characteristic that allows the MOF–SEIRA platform to detect multigases at low concentration (ppm level).

### 2.3 Material Characterization and Thickness Analysis of MOF–SEIRA Platform

**Figure 3:**
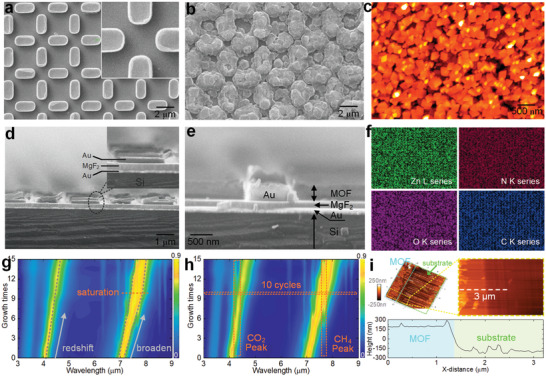
Material characterization and thickness analysis of MOF–SEIRA platform. SEM micrograph showing details of a) the metamaterial absorber and b) the proposed MOF–SEIRA platform. c) AFM micrograph showing details of the MOF–SEIRA platform. d,e) SEM micrograph showing cross‐sectional views of device corresponding to (a,b). Each layer is marked accordingly to indicate the difference. f) EDS mapping analysis of the MOF. g) Deposition times versus spectrum wavelength map showing the effect of MOF thickness on MOF–SEIRA platform (*n* = 15). h) Corresponding map when CO_2_ and CH_4_ with the concentration of 800 ppm are loaded on the platform (*n* = 15). i) AFM micrograph showing the final MOF thickness of MOF–SEIRA platform when MOF deposition times are fixed at 10.

The multiresonant metamaterial absorber was fabricated on a 6 in. silicon wafer by complementary metal–oxide–semiconductor compatible process, i.e., electron beam evaporation, stepper photolithography, and ion beam etch (IBE) (see the Experimental Section). Scanning electron microscope (SEM) images of the metamaterial absorber were shown in **Figure** **3**a. The outline of the pattern was well‐defined, and each layer was tightly bonded and quite distinct from each other (Figure 3d), demonstrating the effectiveness of the fabrication process. After the deposition of MOF on the surface of the nanoarray, the surface morphology of the device changed dramatically, as presented in Figure 3b,e. The atomic force microscopy (AFM) image in Figure 3c also verified the morphology changes caused by the deposition, and the observed rough surface morphology reflected the high surface area of MOF thin film, making it an excellent candidate for gas trapping and sensing. To confirm the composition of the grown ZIF‐8, Fourier transform infrared spectroscopy (FT‐IR) analysis, X‐ray diffraction (XRD) test, and energy dispersive X‐ray spectroscopy (EDS) mapping were performed. The FT‐IR spectrum of the grown ZIF‐8 was consistent with the data in the previously reported literature (Figure S8, Supporting Information).^[^ ^13^ ^]^ According to the FT‐IR spectrum, the strong and broad absorption peak at 1843 cm^−1^ belonging to the N—H bond of dimethylimidazole completely disappeared, meaning that the imidazole links in ZIF‐8 had been fully deprotonated. That is, the prepared film was phase‐pure ZIF‐8. Furthermore, the XRD profile was also in excellent agreement with the test results.^[^ ^18^ ^]^ The peaks at 7.3°, 10.2°, and 12.6° were the primary characteristics related to the ZIF‐8 phase (Figure S9, Supporting Information). The EDS result in Figure 3f verified the presence of Zn, N, C, and O in ZIF‐8, and the distribution of each element was relatively uniform. The Brunauer‐Emmett‐Teller (BET) surface area of the synthesized ZIF‐8 is 1251 m^2^ g^−1^, and its pore volume is 0.608 cm^3^ g^−1^. The CO_2_ uptake of ZIF‐8 is 4.41 mmol g^−1^, which is 4 times higher than the CH_4_ uptake (Note S1, Supporting Information). Collectively, all of the above morphological and compositional analyses demonstrated the successful growth of ZIF‐8 film.

The thickness of MOF thin film is the key factor that influences both platform resonance and gas trapping. Figure 3g shows the MOF growth times versus wavelength map, revealing the effect of MOF thickness on the resonance of MOF–SEIRA platform. A redshift of resonance wavelength and broadening of the spectral peak are clearly observed with the increase of ZIF‐8 thickness. Such redshift is induced by the effective raise of the background dielectric constant. The redshift frequency ∆*ω* can be expressed by the following equation^[^ ^19^ ^]^

(4) $$\begin{array}{c} \frac{\Delta \omega }{\omega_{0}}=-\frac{1}{2}\frac{{\int_{0}^{h}E\cdot (\varepsilon_{\mathrm{ZIF}}-1)\cdot EdV}_{\mathrm{ZIF}}}{\int_{0}^{\infty }{\left|E\right|}^{2}dV} \end{array}$$

where *ω*_0_, **E**, *ε*_ZIF_, *V*_ZIF_, and *V* represent the original resonant frequency, near field, permittivity tensor of ZIF‐8, the volume of ZIF‐8 film, and the full mode volume of the platform resonance, respectively. Since the electric field is mainly perpendicular to the antenna surface, Δ*ω*/*ω*_0_∝(*ε*_ZIF_ − 1) · *h*/*l*_ZIF_, where *ε*_ZIF_ is a constant and *l*_ZIF_ is representative of surface‐averaged localization lengths. Therefore, the redshift frequency ∆*ω* is positively correlated with thickness *h*. As for the broadening phenomenon, it is generally caused by a characteristic mode splitting attributed to the coupling between the resonance of the bare absorber and the vibrational mode of ZIF‐8. It can be estimated via^[^ ^20^ ^]^

(5) $$\begin{array}{c} E_{\pm }=\frac{E_{A}+E_{\mathrm{MOF}}}{2}\pm \sqrt{\frac{{\left(E_{A}-E_{\mathrm{MOF}}\right)}^{2}}{4}+\xi^{2}} \end{array}$$

where *E*_A_ and *E*_MOF_ are the energies of absorber resonance and MOF vibrational mode, respectively, and *ξ* is the coupling strength between them. According to Equation (5), the coupling energy *ξ* leads to a mode splitting between the two modes and thereby causes broadening of the spectral peak. Such broadening becomes severe with increasing thickness until saturation. To further investigate the optimal thickness, the MOF–SEIRA platform is exposed to an environment where CO_2_ and CH_4_ (both 800 ppm) coexist, and the results are shown in Figure 3h. The peaks representing the vibrational modes of CO_2_ and CH_4_ are clear in the map, and platform resonances pass through the peaks as thickness increases. The thickness grown through 10 cycles is determined as the optimal value because the platform resonances at this configuration overlap well with the vibrations of both CO_2_ and CH_4_. The MOF thickness after 10 cycles grown is around 400 nm according to the AFM imaging of the cross‐sectioned film (Figure 3i and the Experimental Section).

### 2.4 Simultaneous Sensing of Greenhouse Gases at Steady State

**Figure 4:**
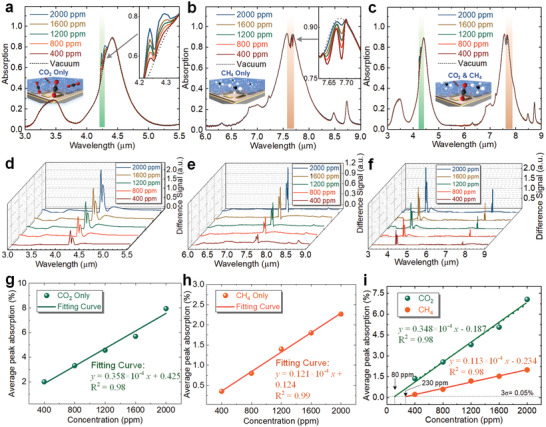
Steady characteristics of the proposed MOF–SEIRA platform for simultaneous sensing of CO_2_ and CH_4_. a) The measured spectra of MOF–SEIRA platform when CO_2_ or b) CH_4_ concentrations change from 0 to 2000 ppm. The insets show the zoomed in spectral change at the CO_2_ or CH_4_ absorption peak. c) The measured spectra when CO_2_ and CH_4_ are simultaneously loaded on the platform. d–f) Corresponding difference signal showing the change in absorption spectra in (a–c). The spectrum obtained by the platform in vacuum was set as the reference spectrum. g–i) Differential absorption versus concentration profile showing their linear relationship. *p* < 0.05, not significant. The sensitivity *S* is defined by the slope angle of the linear fitting curve.

Simultaneous sensing of greenhouse gases at steady state is a critical characteristic for MOF–SEIRA platform. **Figure** **4**a shows the CO_2_ sensing behavior of the MOF–SEIRA platform when the CO_2_ concentration was varied from 0 to 2000 ppm. The variation in the spectral amplitude due to the stretching of C=O is extracted by setting the spectrum obtained in vacuum as a reference, as depicted in Figure 4d. Furthermore, the extracted difference signal is converted into average peak absorption representing the gas vibrational consumption via integrating and averaging (see the Experimental Section). The intensity of the average peak absorption rises continuously as the CO_2_ concentration increase, as shown in Figure 4g. When the sensitivity is defined by the angle of the fitted curve, it reaches 0.0358‰ ppm^−1^. Similar experiments were conducted to investigate the CH_4_ sensing behavior of the MOF–SEIRA platform, as plotted in Figure 4b,e,h. The results reveal that the sensitivity for CH_4_ (0.0121‰ ppm^−1^) is lower than that for CO_2_, which is determined by both the oscillator strength of vibrations and the absorption intensity of the gas in MOF. Further analysis reveals that the sensitivity is more affected by absorption intensity of MOF (Note S8, Supporting Information). Notably, completely reversed behaviors of the change in spectral amplitude was observed, i.e., obvious peaks were produced by CO_2_ and distinct dips by CH_4_, as shown in the insets in Figure 4a,b. It is attributed to the difference in the external to intrinsic damping rates of the two modes of MOF–SEIRA platform corresponding to CO_2_ and CH_4_. This difference has been proven to produce various couple effects, including undercoupled, critically coupled, and overcoupled case.^[^ ^21^ ^]^

Figure 4c,f,i shows the simultaneous sensing of CO_2_ and CH_4_ via the MOF–SEIRA platform. The results indicate that the sensitivity of platform is slightly reduced when compared with the separate measurements of CO_2_ and CH_4_, and the values are 0.0348‰ ppm^−1^ (CO_2_) and 0.0113‰ ppm^−1^ (CH_4_), respectively. The reduction in sensitivity verifies the interference between CO_2_ and CH_4_ due to the adsorption competition in the MOF cavity. However, it is only a small change for the entire measurement in wide concentration range, ≈2.79% of original sensitive for CO_2_ and 6.61% for CH_4_, which benefits from the distribution of CO_2_ and CH_4_ in different areas of the MOF cavity. Specifically, CH_4_ is in closer proximity to the pore windows of MOF than CO_2_.^[^ ^22^ ^]^ Furthermore, the interference can be reduced or even eliminated by a specially designed MOF with independent pores of a suitable size for both CO_2_ and CH_4_. In the experiment, we also notice that CO_2_ is less affected than CH_4_ in the sensing measurement. This is because although CO_2_ competes with CH_4_ in the ZIF‐8 cavity, it is completely independent in the pore aperture (3.4 Å) of ZIF‐8. Such independence is an advantage that makes MOF a potential candidate for multigas sensing applications.

### 2.5 Dynamic Sensing Characteristics of MOF–SEIRA Platform

**Figure 5:**
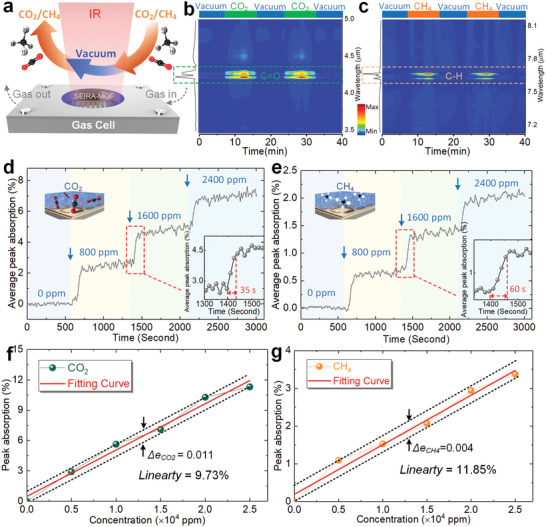
Dynamic characteristics of the proposed MOF–SEIRA platform for simultaneous sensing of CO_2_ and CH_4_. a) Scheme showing dynamic sensing behavior of the proposed MOF–SEIRA platform. b) Time‐resolved difference signal profile of 4.25 µm band, showing the absorption and detection of CO_2_ with concentrations varying from 0 to 1200 ppm repeatedly. The bright band reflects the presence of the asymmetrical stretching mode of C=O=C. c) Time‐resolved difference signal profile of 7.66 µm band. Presence of the *υ*_4_ vibration is indicated accordingly in the bright band. d) Dynamic behavior of MOF–SEIRA platform as CO_2_ and e) CH_4_ concentrations increase from 0 to 2400 ppm in 400 ppm increment (*n* = 200). The inset is an enlarged view of the dotted frame area, showing a response time of 35 s for CO_2_ and 60 s for CH_4_. The arrows in (d,e) represent the inflow of CO_2_ or CH_4_ at the corresponding concentration. f) Linear characteristics of MOF–SEIRA platform for a wide range of CO_2_ and g) CH_4_. Fitting curve for CO_2_: *y* = 4.57 × 10^−6^ *x* +0.49 × 10^−6^ with *R* ^2^ = 0.98. *p* < 0.05, not significant. Fitting curve for CH_4_: *y* = 1.31 × 10^−6^ *x* +0.19 × 10^−6^ with *R* ^2^ = 0.98. *p* < 0.05, not significant.

As a critical indicator in evaluating sensing performance, dynamic characteristics of sensing platform reveals its response time and hysteresis. Therefore, we further investigated the dynamic behavior of MOF–SEIRA platform by placing it in a gas flow cell connected to mass flow controllers (MFC)‐controlled gas sources (see the Experimental Section). The spectral response of platform in consecutive CO_2_/CH_4_−vacuum cycles was then recorded, as depicted in **Figure** **5**a. Figure 5b shows the obtained time‐resolved SEIRA difference signal map. A broad and bright band centered at 4.25 µm appeared rapidly in the signal map with the increase of CO_2_ exposure (maximum 1200 ppm). Furthermore, this bright band decreased until it completely disappeared once the gas was evacuated to a vacuum. Appearance and disappearance of bright band near 4.25 µm band were repeated periodically when MOF–SEIRA platform undergoes consecutive cycling between CO_2_ inflow and vacuum evacuation. Such demonstration indicates that the sorption and desorption of CO_2_ gas is reversible in a vacuum environment. Further investigation reveals that the spectral response of MOF–SEIRA platform is still reversible when it undergoes consecutive cycling between CO_2_ loading and removal (Figure S10, Supporting Information). Analogously, we also observed a bright band centered at 7.66 µm caused by the *υ*_4_ vibrational mode of CH_4_, as shown in Figure 5c. The intensity of the bright band for CH_4_ is slightly weaker than that of CO_2_, because the amount of CH_4_ adsorbed in ZIF‐8 is 4 times less than that of CO_2_ in ZIF‐8 (Figure S1, Supporting Information).

In addition to the reversibility, we also investigated the dynamic response time of the platform by increasing the concentration of CO_2_ and CH_4_ from 0 to 2400 ppm (in 400 ppm increment). The spectral response of the platform was recorded every 15 s interval, and time‐resolved average peak absorption of CO_2_ and CH_4_ was calculated, as shown in Figure 5d,e. The absorption signals kept flat when the platform was positioned within a N_2_ flow, and small fluctuations of the signal originate from the noise of the optical measurement system. After each increase of CO_2_ and CH_4_ concentration, a marked rising can be observed and then fluctuated around the same level. The insets in Figure 5d,e are enlarged views of the dotted frame area showing the evolution of average peak absorption from the rising state to the steady state. When the response time of platform is defined as the differential absorption reaching 95% of the maximum absorption, the response time is estimated around 35 s for CO_2_ and around 60 s for CH_4_. Such difference is due to the larger diffusion coefficient of CO_2_ in ZIF‐8 than CH_4_.^[^ ^23^ ^]^ Notably, there was a delay between the concentration change in gas control module and the optical response of the MOF–SEIRA platform (see arrows in Figure 5d,e). This delay is due to the time it takes for CO_2_ and CH_4_ with a concentration change to flow into the gas cell.

Importantly, we would like to emphasize that excellent linearity rather than nonlinear saturation could be observed in MOF–SEIRA platform when the concentration changed over a wide range. The linear characteristics were analyzed by exposing the platform to CO_2_ and CH_4_ with a wide range of concentrations (from 0 to 2.5 × 10^4^ ppm in 5000 ppm increment), as illustrated in Figure 5f,g. Obviously, the peak absorption varies linearly with the concentration of CO_2_ and CH_4_, and the relationship between them can be fitted by a linear regression line. To evaluate the linear behavior, the linearity of the platform is calculated, and the value for CO_2_ and CH_4_ is 9.73% and 11.85%, respectively (see the Experimental Section). Compared to sensors with nonlinear output, such linear characteristics is a definite advantage for gas sensing, because linear output can significantly simplify subsequent signal processing systems and reduce the cost and difficulty of commercialization of the technology. Besides, the linearity for CO_2_ sensing is better than that for CH_4_ sensing, which is due to the stronger and more independent adsorption of CO_2_ in MOF than CH_4_, as discussed in the previous section. Although the linearity is not perfect due to the large measuring range, the output of the platform is accurate (maximum error: 1.1% and 0.4% for CO_2_ and CH_4_). The linearity of the platform can be further improved by enhancing the adsorption and independence of CO_2_ and CH_4_ in MOF. In addition, the proposed strategy shows excellent scalability, and it can be extended to more gases detections as need by adding the resonance of SEIRA and developing appropriate MOFs (Figure S12, Supporting Information). The comparative analysis of the performance between the MOF–SEIRA platform and the existing SEIRA‐based gas sensors is summarized in Table S5 (Supporting Information). It can be seen that the MOF–SEIRA platform has strong competitiveness among them, especially the characteristics of multigas sensing make it unique.

## 3 Conclusion

In summary, we have developed a rapid and all‐in‐one gas sensor by integrating porous MOF with metamaterial absorber‐based SEIRA platform for simultaneous on‐chip sensing of greenhouse gases. The SEIRA platform offers maximum local near‐field intensity enhancements over 1500‐fold for both sensing bands. The proposed MOF is demonstrated to be highly selective and reversible for the sorption and desorption of both CO_2_ and CH_4_. By exploiting the near‐field enhancement of SEIRA technique and the selective multiple gases trapping of MOF, the MOF–SEIRA platform achieves simultaneous on‐chip sensing of CO_2_ and CH_4_ with fast response time (< 1 min), high accuracy (maximum error, CO_2_: 1.1%, CH_4_: 0.4%), and excellent linearity in wide concentration range (from 0 to 2.5 × 10^4^ ppm). Importantly, the concept is flexible and can extend to more greenhouse gases detections as need by adding additional bands and developing appropriate MOFs. This work does not only provide a powerful tool for greenhouse gases sensing, but also proves the huge potential of SEIRA and MOF in all‐in‐one, real‐time and on‐chip multigas detection.

## 4 Experimental Section

##### Numerical Simulations

The spectral and near‐field characteristics were calculated using a commercial software package (FDTD Solutions v8.19, Lumerical Inc) based on finite‐difference time‐domain method. To boost the modeling efficiency, periodic boundary condition was utilized to model the periodicity of the metamaterial. The platform was excited by plane wave light sources with elliptically polarization in accordance with that in the infrared microscope system. Power Monitor was placed on the reflection path to obtain the reflection spectrum, and both simulation and measurement in the work were performed in reflective mode. The refractive index of Au and Si were derived from Palik et al., and that of MgF_2_ was taken from Malitson et al. The refractive index of ZIF‐8 used in the simulation was calculated by $\varepsilon_{\mathrm{ZIF}}\left(\omega \right)=\varepsilon_{0}+\sum_{j=1}^{5}\left(\varepsilon_{\mathrm{lorentz}}\omega_{j}^{2}/\left(\omega_{j}^{2}-i\cdot 2\delta_{0}\omega -\omega^{2}\right)\right)$, where the terms involved include vibration frequency *ω_i_*, background relative permittivity *ε*_0_, Lorentz permittivity *ε*_Lorentz_, and Lorentz line width *δ*_0_ (Figure S13, Supporting Information). The absorption of CO_2_ and CH_4_ was simplified to $\varepsilon_{\mathrm{gas}}\left(\upsilon \right)=\varepsilon_{1}+\sum_{k=1}^{2}\left(\varepsilon_{\mathrm{lorentz}}\upsilon_{k}^{2}/\left(\upsilon_{k}^{2}-i\cdot 2\delta_{1}\upsilon -\upsilon^{2}\right)\right)$, where the *υ_i_*, *ε*_1_, and *δ*_1_ is the absorption frequency, background permittivity, and Lorentz line width, respectively. The values of these parameters were determined based on the corresponding absorption spectra. The near‐field penetration depth is simulated using a 3D frequency domain power monitor. The thin titanium (Ti) adhesion layer was omitted from the simulation.

##### Nanofabrication of MOF–SEIRA Platform

The devices were fabricated using a complementary metal–oxide–semiconductor compatible process. Figure S14 (Supporting Information) illustrated the detailed fabrication process: a) a high‐resistivity (20 000) 6 in. silicon wafer was cleaned and dried for the subsequent use; b) Ti (10 nm) and Au (100 nm) were sequentially deposited on the silicon surface using a magnetron sputtering system. Here the Ti layer functioned as an adhesion layer to enhance the adhesion between the Au layer and the Si substrate; c) 200 nm thick MgF_2_ was deposited on the Au layer by an e‐beam evaporator system; d) Ti (10 nm) and Au (100 nm) were sequentially deposited on the MgF_2_ dielectric layer and then etched by IBE. Photolithography technique was used to pattern the nanoantenna array via a mid ultraviolet stepper; e) 400 nm thick MOF was grown on device surface; f) the ultimately achieved devices were stored in a drying oven before use.

##### Preparation of MOF Thin Film

The ZIF‐8 film was synthesized by combining the Zinc nitrate hexahydrate and 2‐methylimidazole in a methanol solvent. Figure S15 (Supporting Information) depicted the detailed ZIF‐8 synthesis process. The devices were washed successively in acetone and ethanol to thoroughly remove contaminants, followed by nitrogen drying. Then the cleaned device was immersed in a mixture of 10 mL 2‐methylimidazole (2.5 mol L^−1^, methanol solvent) and 10 mL Zinc nitrate hexahydrate (2.5 mol L^−1^, methanol solvent) for 25 min at room temperature. After the immersion, the device was washed with methanol and blown dry with nitrogen flow. The process was repeated for 10 cycles to obtain a 400 nm thick ZIF‐8 thin film. The thickness measurement of ZIF‐8 was performed in Au surface, and the cross section was obtained by cutting the MOF film carefully.

##### FTIR Measurements

Infrared spectral measurements were performed on a FT‐IR spectrometer (IRTracer‐100, Shimadzu) coupled to an infrared microscope (AIM‐900, Shimadzu) with a 0.4 numerical aperture and × 15 objective. The microscope was equipped with a liquid‐nitrogen‐cooled mercury cadmium telluride. The instrument settings used for all spectral measurement included: 4 cm^−1^ resolution, 20 scans coadded, and absorption mode. The IR spectra were collected in a single 100 × 100 µm^2^ array due to the limitation of knife edge apertures. Three methods were adopted in this work to minimize/eliminate the interference from environmental CO_2_ (Note S14, Supporting Information): I) the entire system was sealed in a transparent container during the test, while dry nitrogen was circulated in the container through the configured enclosure to limit interference from CO_2_ and water vapor; II) the device is placed in the gas cell to during the measurement; III) the spectrum measured on a gold mirror was recorded and used as the background spectrum.

##### Gas Measurement Setup and Data Processing

The experimental setup was composed of three modules as shown in Figure S16 (Supporting Information), namely control module, mix module, and sensing module. In the control module, flow rates of N_2_, CO_2_, and CH_4_ were determined by MFC (SAM, Horiba) with 0–10 and 0–1000 sccm flow range. The valve position of MFC was controlled in real time by a computer equipped with LabVIEW. Therefore, the flow rates could be adjusted dynamically in the software as needed. In the mix module, a small box made of polymethyl methacrylate functioned as a mixer to thoroughly mix the gas from the MFC. Meanwhile, commercial gas sensors were used to calibrate the gas concentration in the mixer. The pressure in the mixer was determined by the cooperation between a vacuum pump and a pressure gauge. Notably, in dynamic sensing experiments, the control module was directly connected to the sensing module to improve the dynamic performance of the setup. In the sensing module, light from IR microscope illuminated the device in the gas cell through a barium fluoride window. The gas cell was made of aluminum alloy and could withstand negative and positive pressure. As for the sensing at steady state, the concentrations of CO_2_ and CH_4_ were controlled by MFCs and calibrated by commercial meters, and then passed into the gas cell. After 5 min of exposure to the gas, the platform was measured under IR microscope. The spectrum of MOF–SEIRA platform before gas loading was set as the reference spectrum. The extraction of the differential absorption spectra was performed by subtracting the reference spectrum from the measured spectrum after gas loading, which can be expressed as *D* = |*A* − *A*_reference_|. The average peak absorption intensity of CO_2_ and CH_4_ was calculated by $A_{p}=1/\left(\lambda_{2}-\lambda_{1}\right)\int_{\lambda_{1}}^{\lambda_{2}}\left(A_{CO_{2},CH_{4}}\right)d_{\lambda }$, where *λ*_1_ and *λ*_2_ are the beginning and end wavelength of average interval, and *A*_CO2,CH4_ is the corresponding differential spectrum of CO_2_ and CH_4_. Analogously, the peak absorption intensity was calculated by $P=\int_{\lambda_{1}}^{\lambda_{2}}\left(A_{CO_{2},CH_{4}}\right)d_{\lambda }$. The linearity was obtained by *δ*_L_ = Δ*e*/FS × 100%, where Δ*e* represents the maximum error in the full scale FS.

##### Apparatus

Scanning electron microscope analyses were obtained using a field emission scanning electron microscope (Carl Zeiss ΣIGMA 500, Germany). The roughness analyses were performed by a commercial atomic force microscopy (Dimension Icon, Bruker Inc). Optical analyses were imaged by an optical microscope (Motic china group CO., Ltd. China). The XRD were obtained by a Ragiku Smartlab Diffractometer using Cu K*α* radiation. Infrared spectral measurements were performed on a FT‐IR spectrometer (IRTracer‐100, Shimadzu) coupled to an infrared microscope (AIM‐900, Shimadzu). EDS analysis was carried out using a Bruker Quantax EDS system with an XFlash Silicon Drift Detector. Photolithography was performed by a Nikon I‐line stepper NSR‐2205 i‐12D. A magnetron sputtering system (FHR. Micro. 200, FHR Inc) was used to deposit Au and Ti layers. BET surface area, CO_2_ and CH_4_ adsorption isotherms were measured by using a volumetric adsorption analyzer (Micromeritics ASAP 2020, USA).

##### Statistical Analysis

Each spectral data are the average of 20 scans. Preprocessing of spectral data, including conversion of wavenumber to wavelength, normalization, and baseline calibration, was carried out by using the software LabSolution (Shimadzu Corporation, Japan). Conversion of wavenumber (*w*_n_) to wavelength (*w*_l_) was performed by *w*_l_ = 10 000/*w*_n_. The near‐field intensity in Figures S3 and S4 (Supporting Information) was normalized by the field strength on the upper surface of the antenna. The average peak absorption for CO_2_ measurement was calculated on the basis of measured data in the range of 4.19–4.34 µm, and that for CH_4_ was calculated in the range of 7.58–7.73 µm. Linear regression was used for curve fitting and modeling the relationship between gas concentration and absorption. Statistics were performed using the software Origin (OriginLab Corporation, USA).

## Conflict of Interest

The authors declare no conflict of interest.

## Supporting information

Supporting Information
