# Supplementary material for: Metal–Organic Framework‐Surface‐Enhanced Infrared Absorption Platform Enables Simultaneous On‐Chip Sensing of Greenhouse Gases
Source: Adv Sci (Weinh). 2020 Sep 18;7(20):2001173. doi: 10.1002/advs.202001173 (PMC7578855; doi:10.1002/advs.202001173)
Supplement: Supplementary file 1 — Supporting Information [file ADVS-7-2001173-s001.pdf]

© 2020 Wiley-VCH GmbH

ADVANCED SCIENCE
Open Access

Supporting Information

for *Adv. Sci.*, DOI: 10.1002/advs.202001173

Metal-Organic Framework-Surface-Enhanced Infrared Absorption Platform Enables Simultaneous On-Chip Sensing of Greenhouse Gases

Hong Zhou, Xindan Hui, Dongxiao Li, Donglin Hu, Xin Chen, Xianming He, Lingxiao Gao, He Huang, Chengkuo Lee, and Xiaojing Mu**

Supporting Information

Metal-Organic Framework-Surface-Enhanced Infrared Absorption Platform Enables Simultaneous On-Chip Sensing of Greenhouse Gases

Hong Zhou, Xindan Hui, Dongxiao Li, Donglin Hu, Xin Chen, Xianming He, Lingxiao Gao, He Huang, Chengkuo Lee and Xiaojing Mu**

Table of Contents

Supplementary Notes 4
Note S1. Structure and adsorption isotherm of ZIF-8 4
Note S2. Analysis of Near-field Intensity Enhancements 5
Note S3. Near-Field Penetration Depth of the SEIRA Platform 7
Note S4. General Resonance Model for MOF-SEIRA Platform 10
Note S5. Infrared Absorption Characterization of MOF Growth 17
Note S6. X-ray Diffraction (XRD) Analysis of ZIF-8 in MOF-SEIRA Platform 18
Note S7. Reversibility Analysis of MOF-SEIRA platform 19
Note S8. Sensitivity analysis for CO_2 and CH_4 sensing 20
Note S9. Scalability Analysis of MOF-SEIRA Platform 22
Note S10. Dielectric Properties of MOF for Simulation 24
Note S11. Fabrication Process of MOF-SEIRA Platform 25
Note S12. Growth of ZIF-8 on the Metamaterial Absorber 26
Note S13. Measurement Setup 27

Note S14. Methods of minimizing the interference from environmental CO_2 and water.......28

Note S15. Performance Comparison of SEIRA-Based Gas Sensors 29

Reference..30

Supplementary Notes

Note S1. Structure and adsorption isotherm of ZIF-8

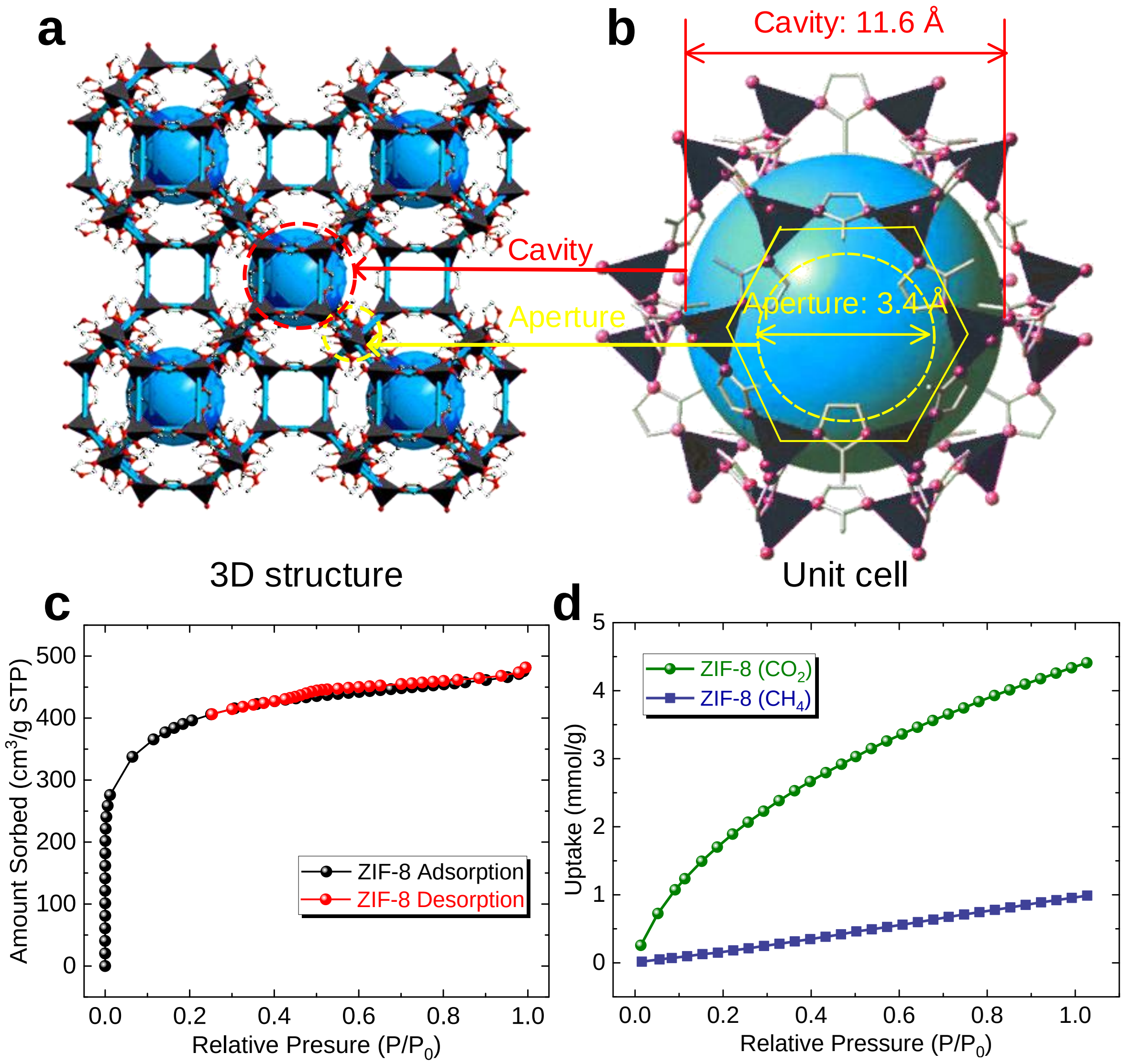

Figure S1. Schematic of MOF showing the details of pore aperture and cavity aperture in a) tetrahedral coordination structure and b) unit cell. c) N_2 adsorption-desorption isotherms for the synthesized ZIF-8 at 77 k. d) CO_2 and CH_4 adsorption isotherms for ZIF-8 samples at 298 K.

The ZIF-8 consists of Zn^{2+} atoms linked to imidazolate anions via nitrogen and assumes tetrahedral coordination. The tetrahedral coordination structure forms nets in the ZIF-8. The

largest cavity (blue pore in **Figure S1**a) in the nets of the ZIF structure is 11.6 Å, and the cavity is connected through small apertures (3.4 Å, formed by the encompassment of six-membered ring window), as shown in Figure S1b. Figure S1c shows the N_2 adsorption-desorption isotherm for the porous ZIF-8. It is a type I isotherm, and the BET surface area (S_{BET}) of the synthesized ZIF-8 is calculated to be 1251 m^2/g by applying the Brunauer-Emmett-Teller equation. Its total pore volume of pores is 0.608 cm^3/g. Figure S1d shows the CO_2 and CH_4 adsorption isotherms at a broad range of high pressures at 298 K. The CO_2 uptake of ZIF-8 is 4.41 mmol/g, which is 4 times higher than the CH_4 uptake.

Note S2. Analysis of Near-field Intensity Enhancements

As shown in **Figure S2**a and S2b, the metamaterial absorber consists of metasurface layer, dielectric spacer, and ground plane. When light enters this type of absorber, high absorption or even perfect absorption occurs. The propagation and multiple reflection of incident light in the absorber is shown in Figure S2c. The absorption is calculated as A = 1- R –T, where R and T represent reflection and transmission, respectively. T is nearly zero across the entire frequency range due to the metallic ground plane which is thicker than the penetration depth of light in the IR range, and then the calculation function becomes A = 1 – R. Therefore, the absorption characteristics of absorbers are studied by reflection spectroscopy.

The near-field distribution of metamaterial absorbers and metasurface arrays is simulated in FDTD Solutions while setting their pattern dimensions to the same. The results show that the metamaterial absorber provides a 2500-fold maximum local near-field intensity enhancement, which is about 10 times higher than that provided by metasurface arrays, as shown in Figure S2d – S2i. The enhancement is affected by the contribution of patterned antenna to metamaterial absorbers / metasurface arrays. The patterned antenna in the metamaterial layer is a type of electric ring resonator (ERR). [1] For metasurface arrays, the incident light is irradiated on the ERR, and then reflected and transmitted. However, for metamaterial absorber, the incident light is reflected multiple times between the ERR and the ground plane: at the air-spacer interface with

ERR, the incident light is partially reflected back to air and partially transmitted into the spacer. The latter continues to propagate until it reaches the ground plane. After the reflection at the ground plane, partial reflection and transmission occur again at the air-spacer interface with ERR. This multiple reflection in the absorber increases the number of interactions between light and ERR and improves their coupling strength, allowing ERR to concentrate more energy. Therefore, the metamaterial absorber provides a higher near field than metasurface arrays.

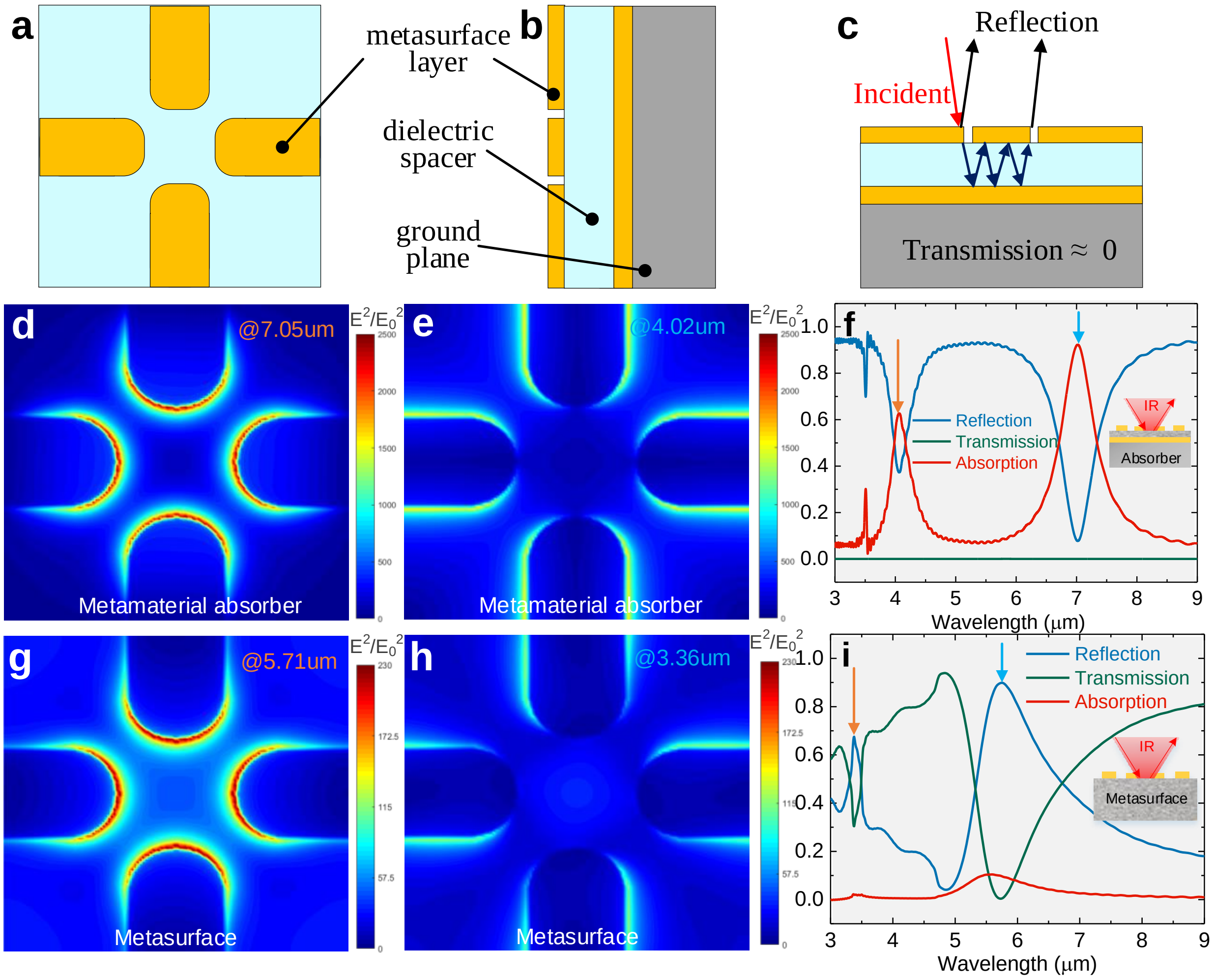

Figure S2. a) Top and b) Side view of the unit cell of the absorber. c) Schematic of incident light propagating in the absorber. d,e) Near-field distribution of the multi-resonant metamaterial absorber. The maximum local near-field intensity enhancements are 2500 times. Simulated spectra of f) the metamaterial absorber and i) the patterned antenna array: Reflection (blue line), transmission (green line), and absorption (red line). g,h)

Near-field distribution of the multi-resonant metasurface. The maximum local near-field intensity enhancements are 230 times.

Note S3. Near-Field Penetration Depth of the SEIRA Platform

Figure S3 investigated the near-field penetration depth of the SEIRA platform by probing the spatial field intensity in the vicinity of the nanoantennas. The red and black curves described the intensity changes of the red and black region (see the inset of Figure S3), which correspond to the two resonances for CO_2 and CH_4, respectively. Clearly, both the field strengths dropped first, then rose, and decayed rapidly after passing the nanoantenna thickness. The SEIRA platform can access up to a 250 nm depth with 10% of the near-field peak intensity, which is orders of magnitude deeper than the penetration depth achieved by surface-enhanced Raman spectroscopy (1-2 nm).[2]

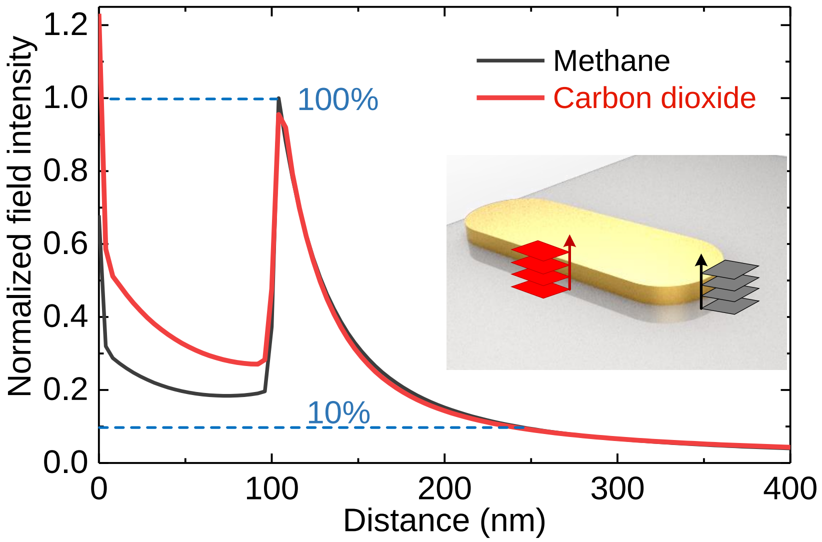

Figure S3. Simulated near-field intensity for the SEIRA platform as a function of the distance to the surface of the corresponding areas of nanoantennas. The near-field intensity is normalized to the field strength on the upper surface of the antenna. The red and black curves correspond to the intensity changes at the red and black planes in the inset, respectively.

Figure S4 investigates the relationship between the penetration depth and the absorber geometry. The analysis is divided into four groups: I) the absorber with initial geometry (marked as Group A), II) changing length dimension *L* (marked as Group B), III) changing pattern (marked as Group C), and IV) changing the thickness of the dielectric layer (marked as Group D). The maximum electric field strength and penetration depth obtained from Figure S4 is summarized in **Table S1**. The following conclusions can be drawn from Table S1:

I) The electric field strength is related to the absorber geometry. The order of electric field strength is: Group C > Group A > Group B > Group D.

II) The penetration depth is independent of the absorber geometry. Although the electric field strength is affected by the absorber geometry, the penetration depths of the four groups are the same and all are 240 nm. This is because the attenuation of the electric field is determined by the dielectric loss of the medium. Therefore, absorbers with different geometries in the same medium have the same penetration depth.

III) The field strength at the maximum penetration depth is related to the absorber geometry. The order of field strength at 240 nm (maximum depth) is: Group C > Group A > Group B > Group D, which is consistent with the order in (I). The results reveal that although the geometry is independent of the absorber geometry, the change of geometry affects the field strength and the performance of the absorber.

Table S1. Summary of electric field strength and penetration depth

	Maximum electric field strength (E^2/E_0^2)	Penetration depth* (nm)	Electric field strength at 240 nm
Group A	2237	240	89.136
Group B	1936	240	70.744
Group C	3881	240	157.367
Group D	1584	240	53.725

* Penetration depth is defined as the depth at which the intensity attenuation is 90%.

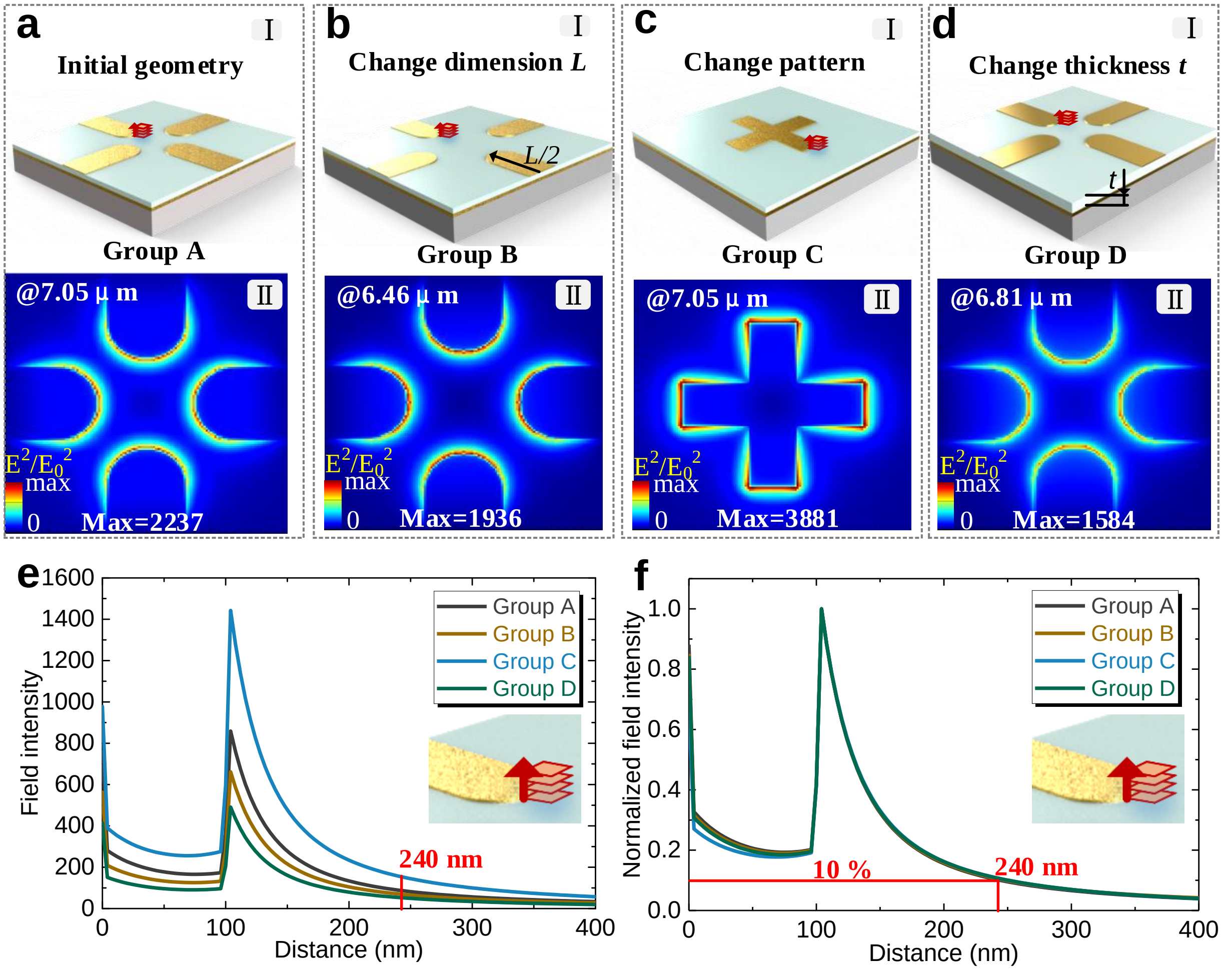

Figure S4. The relationship between the penetration depth and the absorber geometry. Electric field strength of the absorber with a) initial geometry and b,c,d) different geometries. I: Diagram of the metamaterial absorber; II: Top view of electric field distribution. e) Field intensity and f) normalized field intensity of absorbers in a,b,c,d) showing the penetration depth. The inset shows the location of the observation point.

Note S4. General Resonance Model for MOF-SEIRA Platform

The establishment of the general resonance model for MOF-SEIRA platform is based on a temporal coupled mode theory formalism. Since there are three evolutionary processes for the sensing application of MOF-SEIRA platform, the model is built in three steps, namely bare metamaterial absorber, the MOF-SEIRA platform, and the sensing of target gases. The detailed modeling process is discussed as follows:

S4.1. Modeling Bare Metamaterial Absorber

For the metamaterial absorber, the theoretical model consists of a dual-mode (labeled mode A and mode B) optical absorber coupled with two ports representing the incoming (S_{1+}) and outgoing (S_{1-}) radiation (see Fig. 2dII of the main text). Since the dual modes of the absorber (denoted as mode A and mode B) are excited due to the different polarizations of the light source, they are directly coupled to the light source in the model though coupling constants κ_1 and κ_2. The mode can be expressed by:[3]

$$\frac{da_1}{dt} = (j\omega_1 - \gamma_1)a_1 + \kappa_1 S_{1+} - j\cdot\kappa_3 a_2 \tag{S1}$$

$$\frac{da_2}{dt} = (j\omega_2 - \gamma_2)a_2 + \kappa_2 S_{1+} - j\cdot\kappa_3 a_1 \tag{S2}$$

$$S_{1-} = S_{1+} - \kappa_1 a_1 - \kappa_2 a_2 \tag{S3}$$

$$A = 1 - R - T = 1 - \left|r\right|^2 = 1 - \left|\frac{S_{1-}}{S_{1+}}\right|^2 \tag{S4}$$

where a_1 and a_2 are the mode amplitudes of modes, ω_1 and ω_2 the center frequencies, γ_1 and γ_2 the loss rates, κ_3 the coupling constant between mode A and mode B. Since the transmitted wave is limited by the metal bottom layer of the absorber, the transmission T = 0. For time-harmonic field, $da/dt = j\omega t$. Therefore, equation (S1) can be expressed by

$$\left[j(\omega - \omega_1) + \gamma_1\right]a_1 + j\kappa_3 a_2 = \kappa_1 S_{1+} \tag{S5}$$

$$\left[j\left(\omega - \omega_2\right) + \gamma_2 \right] a_2 + j\kappa_3 a_1 = \kappa_2 S_{1+} \tag{S6}$$

Equations (S5) and (S6) can be written in the form of a matrix:

$$\begin{bmatrix} j\left(\omega - \omega_1\right) + \gamma_1 & j\kappa_3 \\ j\kappa_3 & j\left(\omega - \omega_2\right) + \gamma_2 \end{bmatrix} \cdot \begin{bmatrix} a_1 \\ a_2 \end{bmatrix} = \begin{bmatrix} \kappa_1 S_{1+} \\ \kappa_2 S_{1+} \end{bmatrix} \tag{S7}$$

and therefore

$$\begin{bmatrix} a_1 \\ a_2 \end{bmatrix} = \begin{bmatrix} j\left(\omega - \omega_1\right) + \gamma_1 & j\kappa_3 \\ j\kappa_3 & j\left(\omega - \omega_2\right) + \gamma_2 \end{bmatrix}^{-1} \cdot \begin{bmatrix} \kappa_1 S_{1+} \\ \kappa_2 S_{1+} \end{bmatrix} \tag{S8}$$

When we set $Y_i = j\left(\omega - \omega_i\right) + \gamma_i - j\kappa_3$ (i = 1, 2) and solve the set of equations, (S3), (S4) and (S8), the reflection coefficients r can be written as

$$r = 1 - \frac{\kappa_1{}^2 Y_1 + \kappa_2{}^2 Y_2 + j\kappa_3\left(\kappa_1 - \kappa_2\right)^2}{Y_1 Y_2 + j\kappa_3\left(Y_1 + Y_2\right)} \tag{S9}$$

and therefore the absorption A_1 can be expressed by:

$$A_1 = 1 - \left| 1 - \frac{\kappa_1{}^2 Y_1 + \kappa_2{}^2 Y_2 + j\kappa_3\left(\kappa_1 - \kappa_2\right)^2}{Y_1 Y_2 + j\kappa_3\left(Y_1 + Y_2\right)} \right|^2 \tag{S10}$$

An absorption spectrum containing two Lorentzian line shapes can be obtained by plotting equation (S10) in MATLAB software. The resonance parameters determining the line-width and amplitude are extracted by fitting equation (S10) to simulated spectrum (Fig. 2dIII of the main text). The obtained values are listed in **Table S2**. Besides, the independence between mode A and mode B is investigated by varying the coupling constant κ_3 from 0.2π to 200π. The calculated results indicate that the effect of κ_3 on absorption spectrum is negligible when the resonant frequencies of the two modes is significantly different, as plotted in **Figure S5**, theoretically demonstrating the independence between the two modes of the absorber.

Table S2. The extracted values of resonance parameters and comparison of coupling constant.

Comparison	λ_1 (μm)	λ_2 (μm)	γ_1 (×10^{12})	γ_2 (×10^{12})	κ_1 (×10^6)	κ_2 (×10^6)	**κ_3 (×10^6)**
Group A	4.05	7.16	3.31×2π	1.95×2π	0.48×2π	0.48×2π	**2π**
Group B	4.05	7.16	3.31×2π	1.95×2π	0.48×2π	0.48×2π	**200π**
Group C	4.05	7.16	3.31×2π	1.95×2π	0.48×2π	0.48×2π	**0.02π**

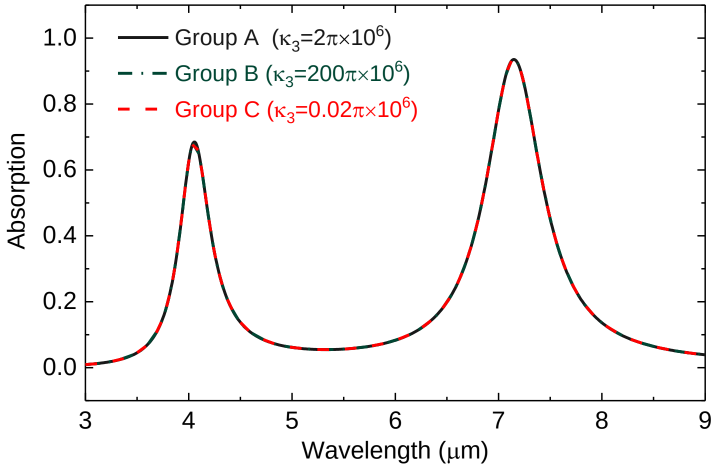

Figure S5. The spectra of metamaterial absorber calculated using the general resonance model with different coupling constants showing the independence between the two modes of metamaterial absorber

S4.2. Modeling MOF-SEIRA Platform

When MOF thin film covers on the surface of absorber to form MOF-SEIRA platform, the above model is extended via the temporal coupled mode theory to include the effects of the MOF thin film. It is achieved by coupling (coefficient μ) a purely dissipative mode representative of the MOF absorption to the absorber model (Fig. 2eII of the main text). According to the measured infrared absorption of MOF, there are five peaks representative of five vibrational modes of MOF in the spectral band of interest, labeled p_1, ..., p_5. According above analysis, mode A and mode B are independent and the coupling constant is ignored. The coupled mode equations for this configuration can be written as:

$$\frac{da_1}{dt} = (j\omega_1 - \gamma_1)a_1 + \kappa_1 S_{1+} \tag{S11}$$

$$\frac{da_2}{dt} = (j\omega_2 - \gamma_2)a_2 + \kappa_2 S_{1+} - j\cdot\mu_B \mathbf{B} \tag{S12}$$

$$\frac{d\mathbf{B}}{dt} = (j\boldsymbol{\omega}_{\mathbf{B}} - \boldsymbol{\gamma}_{\mathbf{B}})\mathbf{B} - j\cdot\boldsymbol{\mu}_{\mathbf{B}} a_2 \tag{S13}$$

$$S_{1-} = S_{1+} - \kappa_1 a_1 - \kappa_2 a_2 \tag{S14}$$

$$A = 1 - R - T = 1 - |r|^2 = 1 - \left|\frac{S_{1-}}{S_{1+}}\right|^2 \tag{S15}$$

where the matrices $\boldsymbol{\omega}_{\mathbf{B}} = \begin{bmatrix} \omega_{b1} \\ \omega_{b2} \\ \omega_{b3} \\ \omega_{b4} \\ \omega_{b5} \end{bmatrix}$, $\boldsymbol{\gamma}_{\mathbf{B}} = \begin{bmatrix} \gamma_{b1} \\ \gamma_{b2} \\ \gamma_{b3} \\ \gamma_{b4} \\ \gamma_{b5} \end{bmatrix}$, $\mathbf{B} = \begin{bmatrix} b_1 \\ b_2 \\ b_3 \\ b_4 \\ b_5 \end{bmatrix}$, and $\boldsymbol{\mu}_{\mathbf{B}} = \begin{bmatrix} \mu_{b1} \\ \mu_{b2} \\ \mu_{b3} \\ \mu_{b4} \\ \mu_{b5} \end{bmatrix}$ represent the center frequencies, loss rates, mode amplitudes, and coupling coefficient of MOF with five vibrational modes, respectively. For time-harmonic field, $d_B / dt = j\omega t$. Therefore, the mode amplitudes can be solved by substituting it into equation (S13).

$$\mathbf{B} = -\frac{j\boldsymbol{\mu}_{\mathbf{B}} a_2}{j(\omega - \boldsymbol{\omega}_{\mathbf{B}}) + \boldsymbol{\gamma}_{\mathbf{B}}} \tag{S16}$$

Substituting equation (S16) into equation (S10), we can obtain:

$$a_2 = \frac{\kappa_2 S_{1+}}{j(\omega - \omega_2) + \gamma_2 + \boldsymbol{\mu}_{\mathbf{B}} \Big/ \left[j(\omega - \boldsymbol{\omega}_{\mathbf{B}}) + \boldsymbol{\gamma}_{\mathbf{B}} \right]} \tag{S17}$$

And then solving the set of equations, (S11)-(S15), in conjunction with equation (S17),

$$A_2 = 1 - \left| 1 - \frac{\kappa_1^{\ 2}}{j(\omega - \omega_1) + \gamma_1} - \frac{\kappa_2^{\ 2}}{j(\omega - \omega_2) + \gamma_2 + \Gamma_1 + \Gamma_2 + \Gamma_3 + \Gamma_4 + \Gamma_5} \right|^2 \tag{S18}$$

where $\Gamma_i = \dfrac{\mu_{bi}^{\ 2}}{j(\omega - \omega_{bi}) + \gamma_{bi}}$ (i = 1, 2, 3, 4, 5) represent the effect of MOF on platform. The

resonance parameters determining the line-width and amplitude are extracted by fitting equation (S18) to simulated spectrum (Fig. 2eIII of the main text). The obtained values are listed in **Table S3**. **Figure S6** shows the spectrum of MOF-SEIRA calculated using the established model. The vibrational modes of MOF thin film can be observed clearly in the spectrum. Besides, when the vibrational mode of the MOF film is close to the resonance of the platform, the signal peak in the spectrum is more distinct.

Table S3. The extracted values of resonance parameters of the extended resonance model.

λ_1 (μm)	λ_2 (μm)	γ_1 ($\times 10^{12}$)	γ_2 ($\times 10^{12}$)	κ_1 ($\times 10^6$)	κ_2 ($\times 10^6$)	κ_3 ($\times 10^6$)	λ_{b1} (μm)
4.41	7.60	3.58×2	1.55×2	0.47×2π	0.47×2	2π	6.33
λ_{b2} (μm)	λ_{b3} (μm)	λ_{b4} (μm)	λ_{b5} (μm)	μ_{b1} ($\times 10^{11}$)	μ_{b2} ($\times 10^{11}$)	μ_{b3} ($\times 10^{11}$)	μ_{b4} ($\times 10^{11}$)
6.92	7.61	8.46	8.71	9.99×2π	8.86×2π	7.86×2π	4.16×2π
μ_{b5} ($\times 10^{11}$)	γ_{b1} ($\times 10^{11}$)	γ_{b2} ($\times 10^{11}$)	γ_{b3} ($\times 10^{11}$)	γ_{b4} ($\times 10^{11}$)	γ_{b5} ($\times 10^{11}$)		
9.86×2π	3.86×2π	6.00×2π	7.26×2π	0.9×2π	4.9×2		

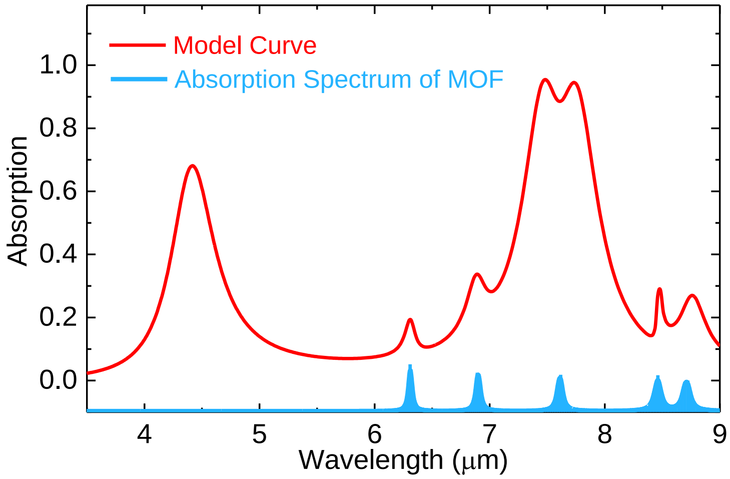

Figure S6. The spectrum of MOF-SEIRA platform calculated using the extended resonance model. The simplified absorption spectra of MOF are shown at the bottom of the panel, and the value is enlarged in scale.

S4.3. Modeling the Sensing Behavior of MOF-SEIRA Platform

When CO_2 and CH_4 are trapped in MOF-SEIRA platform (Fig. 2fI), the model is extended again by introducing couplings (υ_c, υ_e) between the MOF resonance and the dissipative mode representing gas absorption. According to the interaction between gases vibration and platform resonances, CO_2 and CH_4 are coupled to mode A and mode B of the MOF-SEIRA platform, respectively (Fig. 2fII of the main text). The coupled mode equations for this configuration can be written as:

$$\frac{da_1}{dt} = (j\omega_1 - \gamma_1)a_1 + \kappa_1 S_{1+} \tag{S19}$$

$$\frac{da_2}{dt} = (j\omega_2 - \gamma_2)a_2 + \kappa_2 S_{1+} - j \cdot \mu_B b \tag{S20}$$

$$\frac{d\mathbf{B}}{dt} = (j\boldsymbol{\omega}_{\mathbf{B}} - \boldsymbol{\gamma}_{\mathbf{B}})\mathbf{B} - j \cdot \boldsymbol{\mu}_{\mathbf{B}} a_2 \tag{S21}$$

$$\frac{dc}{dt} = (j\omega_c - \gamma_c)c - j\upsilon_c a_1 \tag{S22}$$

$$\frac{de}{dt} = (j\omega_e - \gamma_e)c - j\upsilon_e a_2 \tag{S23}$$

$$S_{1-} = S_{1+} - \kappa_1 a_1 - \kappa_2 a_2 \tag{S24}$$

$$A = 1 - R - T = 1 - \left|r\right|^2 = 1 - \left|\frac{S_{1-}}{S_{1+}}\right|^2 \tag{S25}$$

where c and e are the mode amplitudes of CO_2 and CH_4, ω_c and ω_e the center frequencies, γ_c and γ_e the loss rates, υ_c and υ_e the coupling coefficient. Solving the set of equations, (S19) - (S26), the absorption A_3 can be written as:

$$A_3 = 1 - \left|1 - \frac{\kappa_1^2}{j(\omega - \omega_1) + \gamma_1 + Y_c} - \frac{\kappa_2^2}{j(\omega - \omega_2) + \gamma_2 + \Gamma_1 + \Gamma_2 + \Gamma_3 + \Gamma_4 + \Gamma_5 + Y_e}\right|^2 \tag{S26}$$

where $\Gamma_i = \frac{\mu_{bi}^2}{j(\omega - \omega_{bi}) + \gamma_{bi}}$ (i = 1, 2, 3, 4, 5) and $Y_{c,e} = \frac{\upsilon_{c,e}^2}{j\left(\omega - \omega_{c,e}\right) + \gamma_{c,e}}$. The $Y_{c,e}$ represent

the effect of CO_2 and CH_4 on platform. The values of the model are listed in **Table S4**. **Figure S7** shows the calculated spectrum of MOF-SEIRA of platform with the adsorption of CO_2 and CH_4. We can clearly observe the signal peaks corresponding to gases absorption in the ultimately achieved spectrum after data processing. Notably, there is a slight spectral overlap between the peaks of MOF and CH_4. Since the peak derived from MOF is constant after the fabrication of platform, the spectral change is determined by the vibration of CH_4, which is the basis for the CH_4 sensing.

Table S4. The extracted values of resonance parameters of the extended resonance model.

Parameter	λ_c (μm)	λ_e (μm)	γ_c ($\times 10^{12}$)	γ_e ($\times 10^{12}$)	υ_c ($\times 10^{11}$)	υ_e ($\times 10^{11}$)
Value	4.25	7.66	3.21×2π	2.91×2π	8.21×2π	3.33×2π

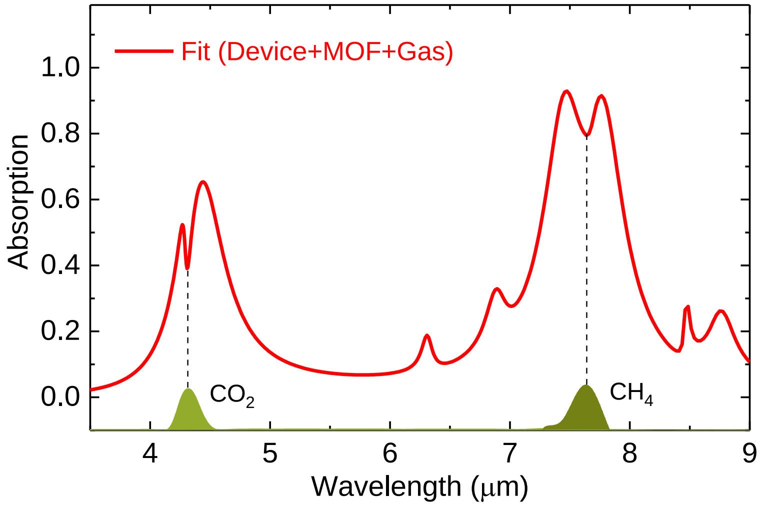

Figure S7. The spectrum of MOF-SEIRA of platform with the adsorption of CO_2 and CH_4 calculated using the extended resonance model. The simplified absorption spectra of CO_2 and CH_4 are shown at the bottom of the panel, and the value is enlarged in scale.

Note S5. Infrared Absorption Characterization of MOF Growth

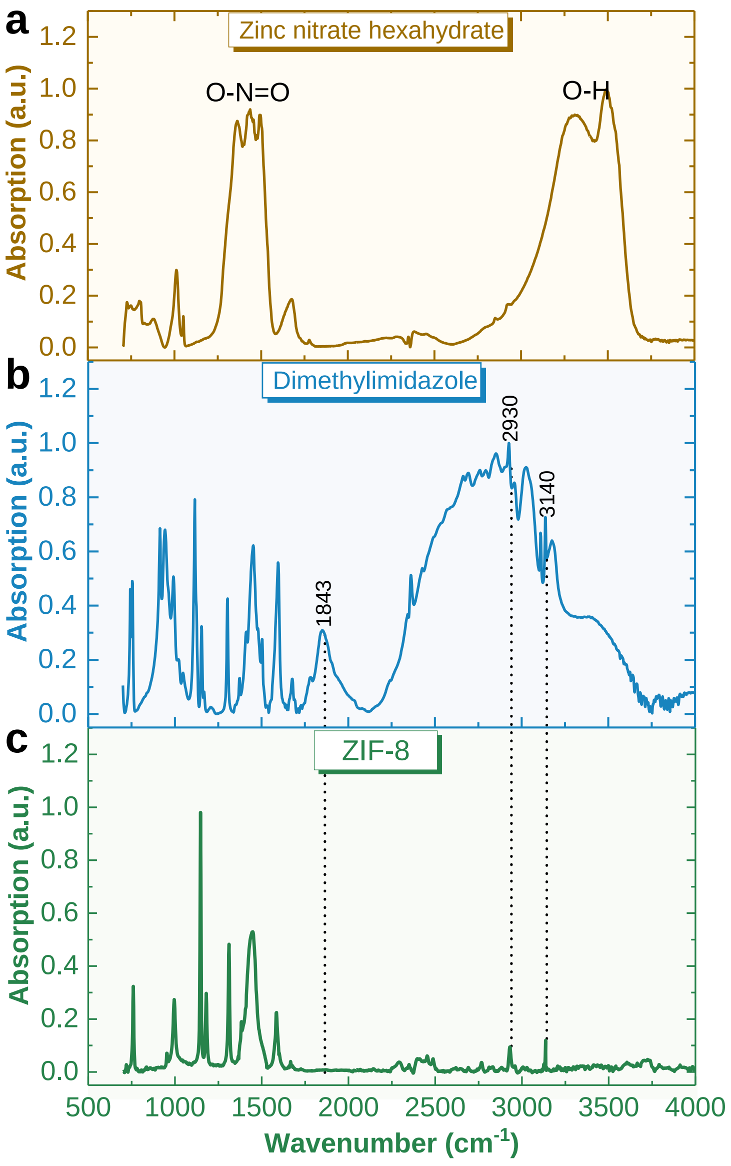

Figure S8. Infrared absorption characterization of MOF synthesis. Measured infrared absorption spectrum of a) zinc nitrate hexahydrate, b) dimethylimidazole, and c) ZIF-8.

Figure S8 shows Fourier transform infrared spectroscopy results of zinc nitrate hexahydrate, dimethylimidazole, and ZIF-8. The peaks at 1591 is ascribed to the bending of N-H. the imidazole ring stretch absorbs with several bands between 1350-1500 cm^{-1}.[4] The peaks from

2500 to 3000 cm^{-1} is ascribed to the stretching vibration of N-H, C-H, and O-H. Clearly, the spectrum of ZIF-8 has many peaks similar to those of dimethylimidazole between 700-1500 cm^{-1}, which is due to the ligands of 2-methylimidazole in ZIF-8.[5] Importantly, the strong and broad absorption peak at 1843 cm^{-1} belonging to the N-H bond of dimethylimidazole completely disappeared, meaning that the imidazole links in ZIF-8 had been fully deprotonated.

Note S6. X-ray Diffraction (XRD) Analysis of ZIF-8 in MOF-SEIRA Platform

Figure S9 depicts the XRD pattern of ZIF-8 with 25 cycles of growth. There is a sharp peak at 2θ = 7.3°, indicating a high degree of crystallinity for the synthesized ZIF-8. Diffraction peaks are mainly located at: 7.3° (011), 10.3° (200), 12.6° (112), 14.6° (022), 16.2° (013), 17.9° (222), 24.4° (233), and 26.6° (134). Clearly, the XRD profile was in excellent agreement with the test results in reference.[6]

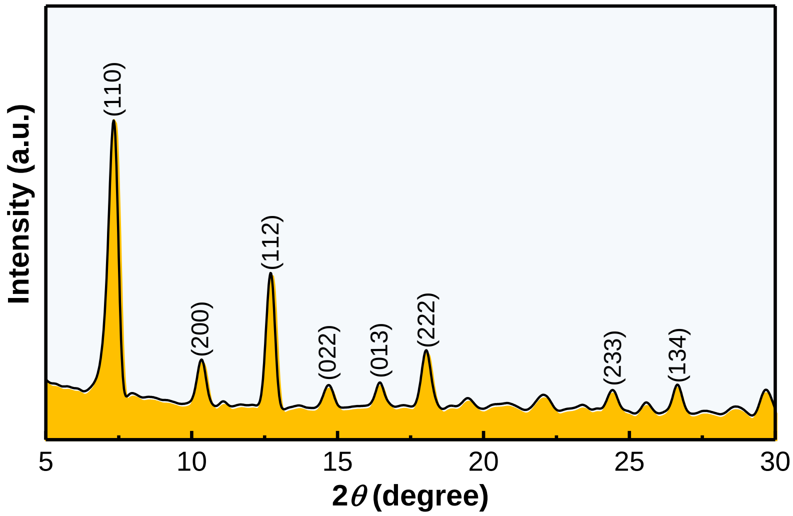

Figure S9. XRD spectra of ZIF-8 showing the phase of ZIF-8.

Note S7. Reversibility Analysis of MOF-SEIRA platform

The reversibility of MOF-SEIRA platform is investigated by cyclically changing the concentration of CO_2 between 0 ppm and 2000 ppm. The experimental results are shown in **Figure S10**. The spectral response is still reversible when the CO_2 concentration changes cyclically between 0 ppm and 2000 ppm. However, its response time (the time to reach a steady state) is relatively long when compared to that in a vacuum environment (Figure 5b and 5c of main text).

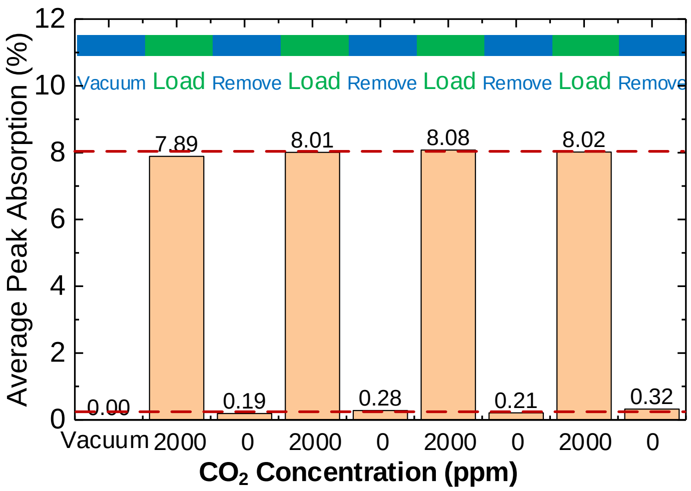

Figure S10. The reversible behavior of the sensor when the CO_2 concentration changes cyclically between 0 ppm and 2000 ppm. N_2 is used as the carrier gas.

Note S8. Sensitivity analysis for CO_2 and CH_4 sensing

The sensitivity of the sensor is determined by both the oscillator strength of vibrations and the absorption intensity of the gas in MOF.

I) *Oscillator strength of vibrations*: The oscillator strength is proportional to the integral of the molar absorptivity (denoted as E_{nm}) over the frequency range covered by the band:[7]

$$E_{nm} = \int \varepsilon(v)\,dv \tag{R1}$$

where v represents the frequency, and the molar absorptivity ε is that in Beer's law:

$$A = \varepsilon c l \tag{R2}$$

where c and l represent the molar concentration and the pathlength, respectively. Substituting Equation (R2) into Equation (R1) gives:

$$E_{nm} = \int \frac{A(v)}{cl} dv \tag{R3}$$

The E_{nm} is related to the oscillator strength f_{nm} by:

$$f_{nm} = 1.441 \times 10^{-18} E_{nm} = 1.441 \times 10^{-18} \int \frac{A(v)}{cl} dv \tag{R4}$$

The absorbance $A(v)$ of carbon dioxide and methane obtained from the database of the National Institute of Standards and Technology, U.S. Department of Commerce.[8] Then, the oscillator strength of CO_2 and CH_4 is calculated as:

$$f_{nmCO2} < f_{nmCH4}$$

II) *Absorption intensity of MOF*: The absorption intensity of ZIF-8 is another factor that affects the sensitivity of the sensor. The absorption intensity of CO_2 and CH_4 in ZIF-8 is shown in Figure S11. The absorption intensity of ZIF-8 to CO_2 is stronger than that to CH_4.

It can be concluded:

- Device sensitivity: CO_2 > CH_4
- Oscillator strength: CO_2 < CH_4
- Absorption intensity: CO_2 > CH_4

In summary, although the oscillator strength of CH4 is stronger than that of CO2, the device sensitivity for CO_2 sensing is still higher than that for CH_4, indicating that the sensitivity is more affected by absorption intensity.

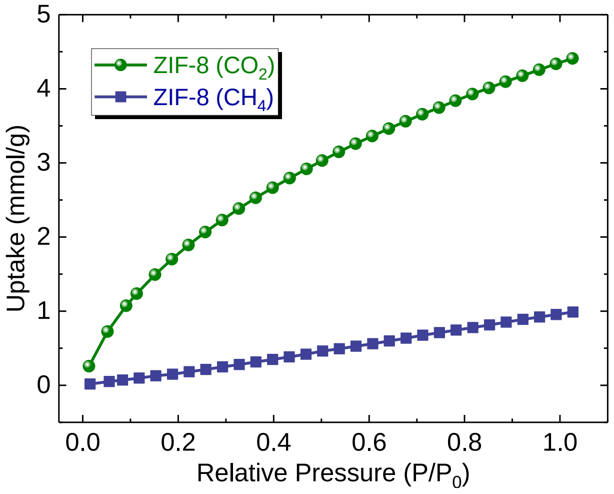

Figure S11. CO_2 and CH_4 adsorption isotherms for ZIF-8 samples at 298 K.

Note S9. Scalability Analysis of MOF-SEIRA Platform

The proposed strategy of integrating MOF and SEIRA for multi-gas sensing is flexible and is not limited to the detection of CO_2 and CH_4. It can be extended to more gases detections as need by adding the resonance of SEIRA and developing appropriate MOFs. In this section, we introduce a possible way to increase the type of gas sensed by the platform. First, it is necessary to design a SEIRA platform with multiple resonance when aiming to detect more gases. Our method for achieving multiple resonances is to add independent resonant units to the metasurface pattern, as shown in **Figure S12**. By adding two sets of nanodipoles to the cross-shaped metasurface, the SEIRA platform can provide triple (Figure S12b) and quadruple (Figure S12c) resonances for multi-gas sensing. In addition, these resonances (labeled as A, B, C, D in Figure S12) are independent of each other (Figure S12aII, S12bII, and S12cII), thereby weakening the mutual interference in the sensing process. Importantly, the resonance can be individually adjusted to suit different gas vibrations, as discussed in section 2.2. Apart from the design of multi-resonant SEIRA platform, it is also essential to develop appropriate MOFs. As a porous materials with hybrid network structures, the mesoporous properties of MOF can be adjusted by the judicious and rational choice of building block molecules. Furthermore, the applicable properties of MOFs can be further improved by controllable encapsulation of nanoentities of functional and morphological features and various MOF topologies, morphologies and composites.[9] It means that the aperture sizes and functional sites of MOFs can be accurately designed and constructed using isoreticular chemistry.[10] For instance, ZIF-L constructed by

alternative assembly of Zn^{2+} ions and 2-methylimidazole linkers shows a selective adsorption of carbon monoxide, toluene, 1,3-butadiene, and tetrachloroethylene.[11] Based on the strategy proposed in this paper, it is expected that the integration of ZIF-L and multi-resonant SEIRA platform shown in Figure S12c can possibly enable the multiplexed sensing of carbon monoxide, toluene, 1,3-butadiene, and tetrachloroethylene. Collectively, it can be concluded that such MOF-SEIRA platform has excellent scalability.

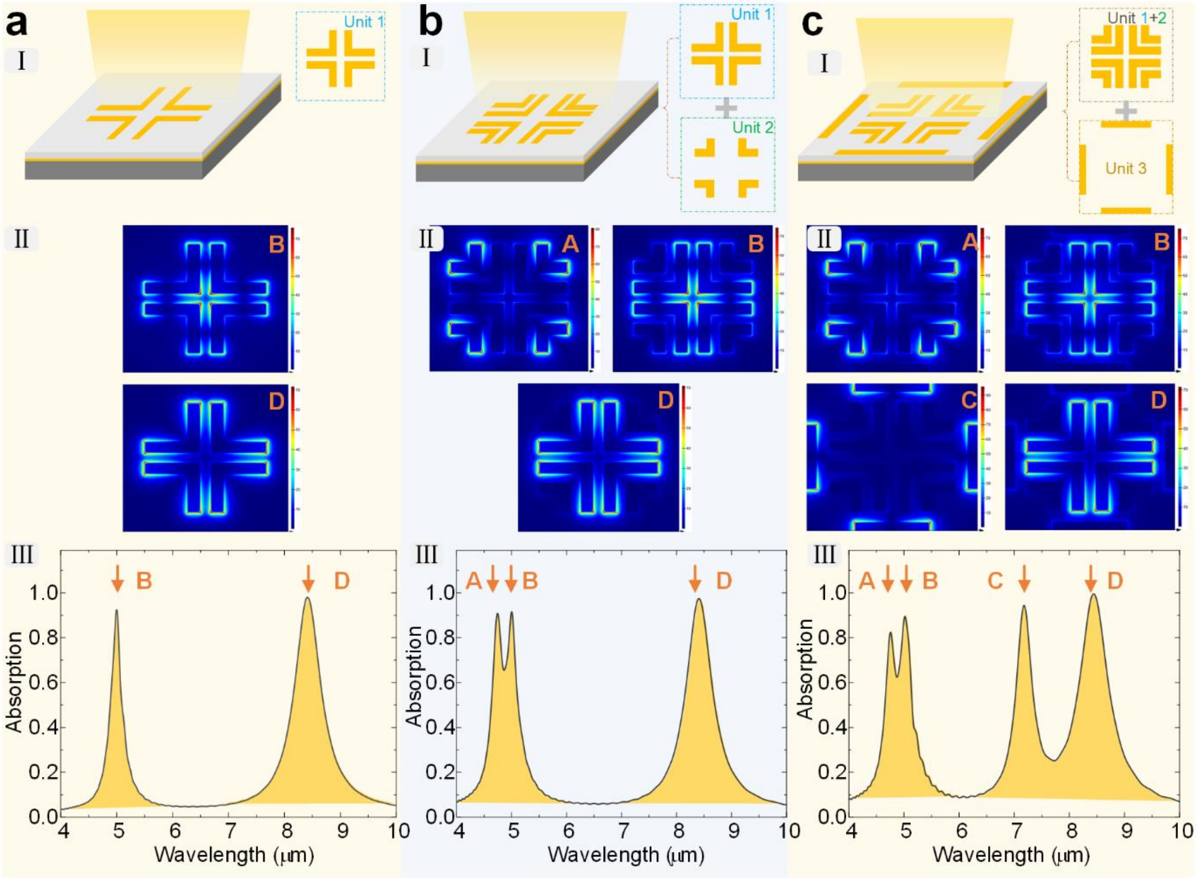

Figure S12. Scalability characteristics of SEIRA platform for multi-gas sensing. a) Dual-band SEIRA platform. b) Tri-band SEIRA platform. c) Quad-band SEIRA platform. I: Schematic of the metamaterial absorber. Inset showing the addition of independent nanodipoles to cross-shaped unit for more resonance. II: Near-field distribution at resonance. III: Simulated absorption spectra.

Note S10. Dielectric Properties of MOF for Simulation

In order to simulate the spectral characteristics of MOF-SEIRA platform, the gas-selective material ZIF-8 is modeled based on the experimentally obtained infrared absorption spectrum. According to the measured infrared absorption of ZIF-8, there are five peaks in the spectral band of interest. Therefore, a Lorentz model with five oscillators corresponding to the experimental data is built using following equation:[12]

$$\varepsilon(\omega) = \varepsilon_0 + \sum_{j=1}^{5} \frac{\varepsilon_{lorentz}\omega_j^2}{\omega_j^2 - i \cdot 2\delta_0\omega - \omega^2} \quad \text{(S27)}$$

Here, ω_i is the Lorentz resonance frequency and ε_0 is the background relative permittivity of MOF, 1.8. The values of the Lorentz permittivity, $\varepsilon_{Lorentz}$, and the Lorentz linewidth, δ_0, have been fixed at 0.003 and 8.0×10^{11} rad/s, respectively. According to the infrared spectrum of ZIF-8, the spectral positions $\lambda = 6\times10^{14}\pi/\omega$, and the five oscillators are fixed at 6.31 µm, 6.9 µm, 7.6 µm, 8.46 µm, and 8.71 µm. Therefore, the dielectric characteristics can be calculated by the Lorentz model, as shown in **Figure S13**.

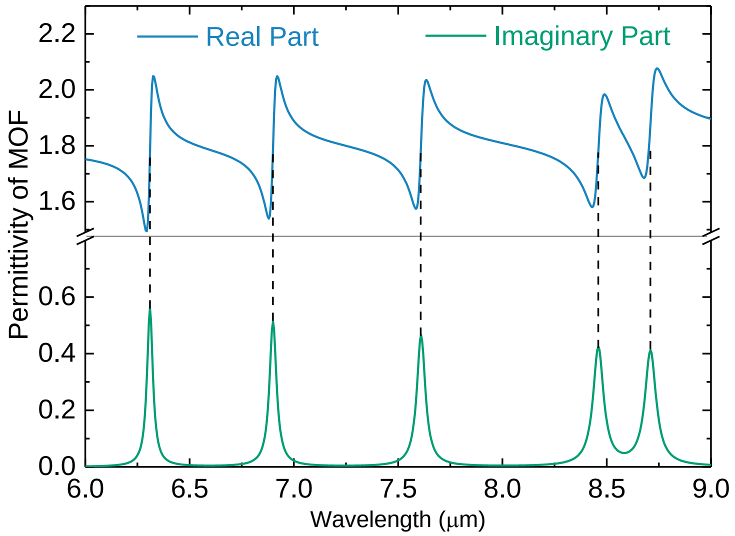

Figure S13. Real (blue curve) and imaginary (green curve) parts of the modeled permittivity function for ZIF-8.

Note S11. Fabrication Process of MOF-SEIRA Platform

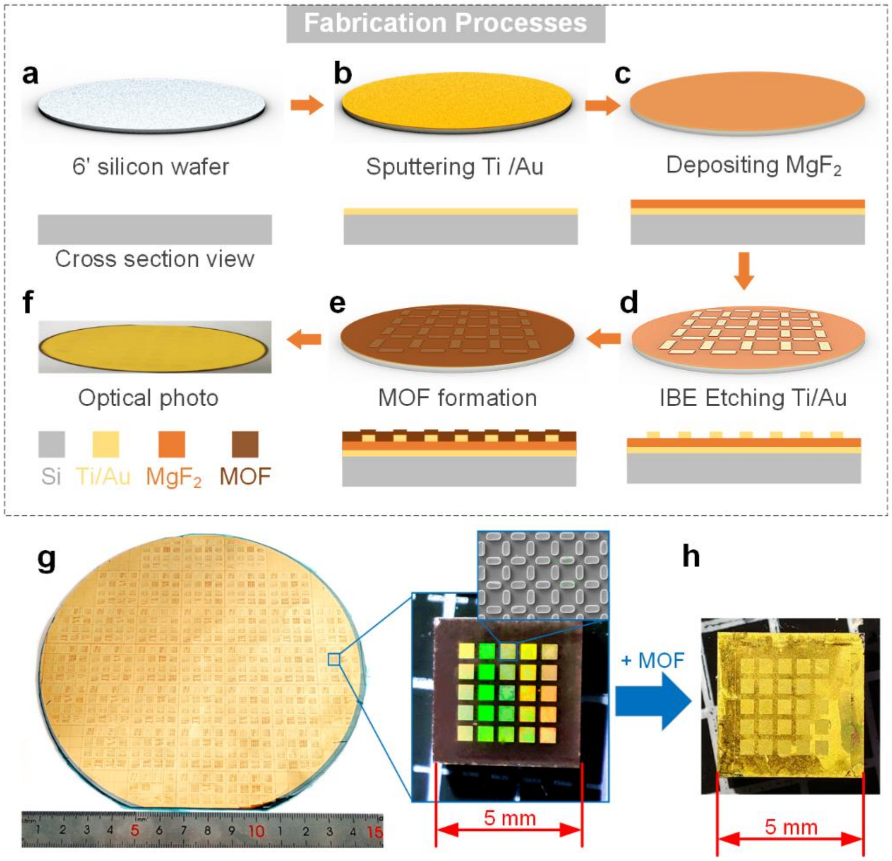

Figure S14. Schematic diagram of processes to fabricate the MOF-SEIRA platform. a) a high-resistivity (20 000) 6" silicon wafer was cleaned and dried for the subsequent use; b) Ti (10 nm) and Au (100 nm) were sequentially deposited on the silicon surface using a magnetron sputtering system; c) 200 nm thick MgF_2 was deposited on the Au layer by an e-beam evaporator system; d) Ti (10 nm) and Au (100 nm) were sequentially deposited on the MgF_2 dielectric layer and then etched by IBE. Photolithography technique was used to pattern the nanoantenna array via a mid ultra violet stepper; e) 400 nm thick MOF was grown on device surface; f) the ultimately achieved devices were stored in a drying oven before use. The optical picture of g) the fabricated absorber and h) the MOF-SEIRA platform.

Note S12. Growth of ZIF-8 on the Metamaterial Absorber

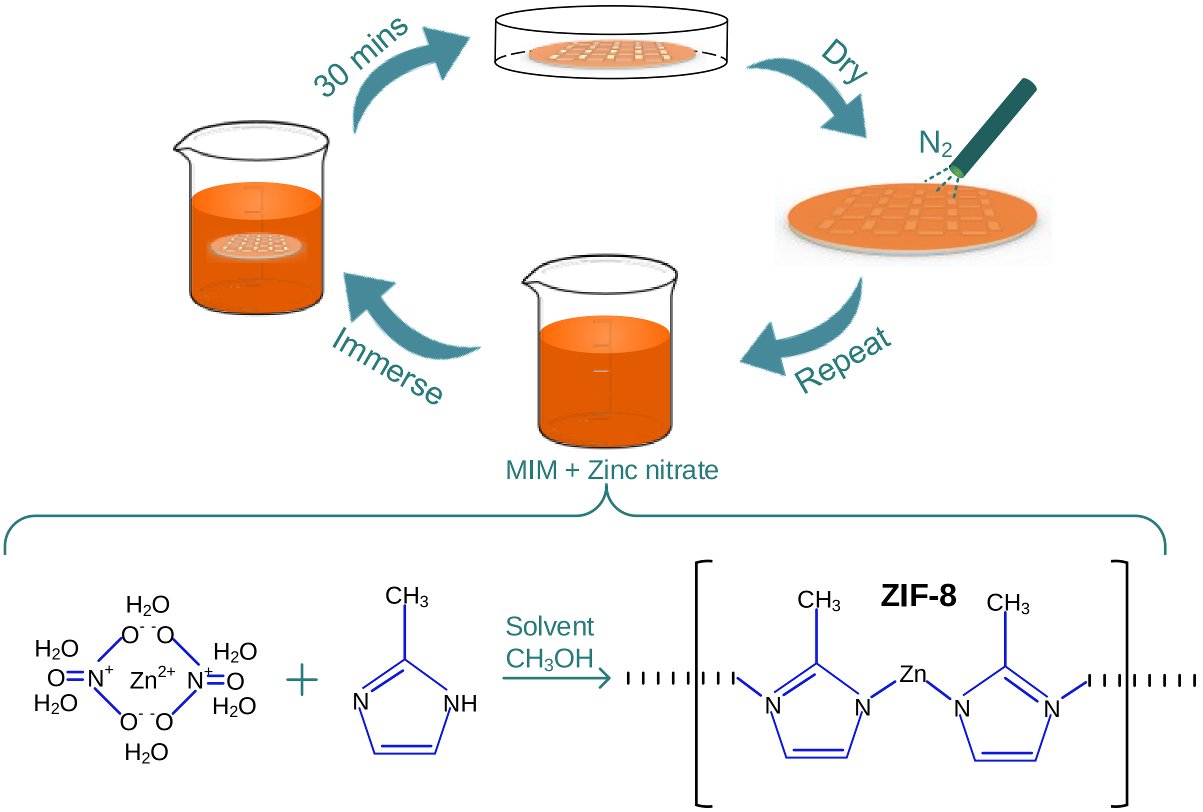

Figure S15. The illustration for the growth of ZIF-8 on the absorber.

Note S13. Measurement Setup

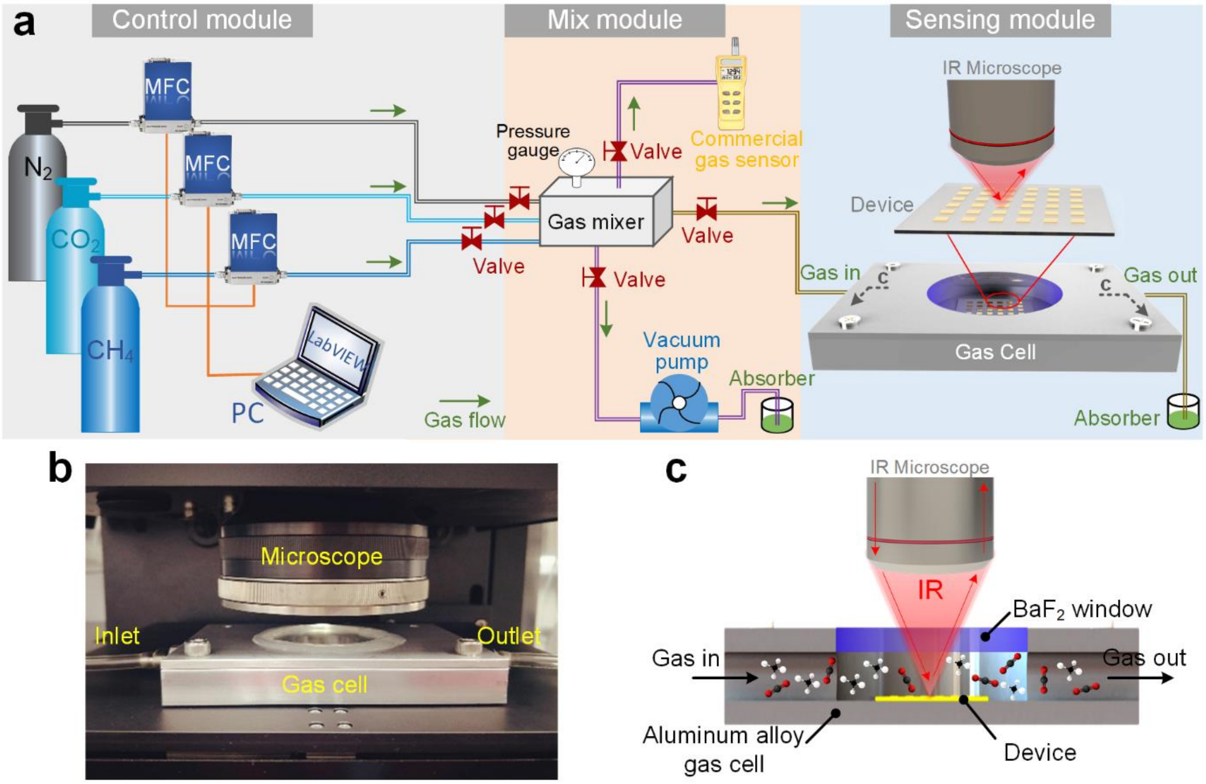

Figure S16. a) Schematic diagram of experimental setup. b) Optical photo and c) schematic illustration showing the sensing module.

Note S14. Methods of minimizing the interference from environmental CO_2 and water

Three methods were adopted in this work to minimize/eliminate the interference from environmental CO_2. I) The whole experimental setup, including the FT-IR spectrometer, the infrared microscope, the fabricated device and gas cell, is wrapped in a flexible, large polyethylene bag (**Figure S17**). Before the measurement, nitrogen gas is poured into the bag for half an hour to reduce the CO_2 and water in the space near the experimental setup. In addition, there are specially designed accessories in the microscope (AIM-900, Shimadzu), through which nitrogen gas continuously flow into the instrument during the measurement process, thereby ensuring that the optical path in the instrument is protected by nitrogen gas; II) The device is placed in the gas cell to during the measurement, as shown in Figure S17 in the supporting information. There are two purposes: (1) to isolate it from the environment to reduce the interference of CO_2 and water; (2) to control the gas concentration in the sensing experiment. III) A background spectrum is run before each measurement of the spectral response of the device. The background spectrum is measured on a gold mirror and contains interference contributions from the instrument and the environments (e. g. CO_2). Therefore, the interference contributions can be eliminated by normalizing the device spectrum against the background spectrum.

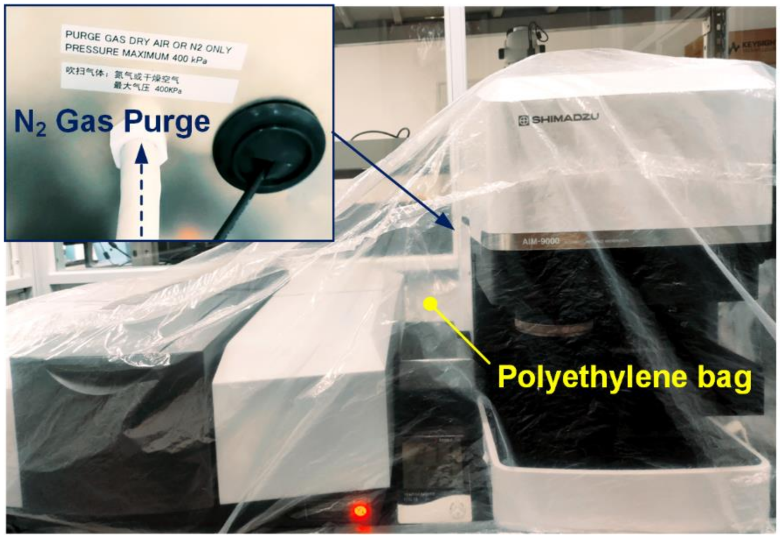

Figure S17. Photo showing the details of the experimental setup wrapped in a bag. Inset: Specially designed accessory of the microscope, through which nitrogen gas continuously flow into the instrument.

Note S15. Performance Comparison of SEIRA-Based Gas Sensors

Table S5. Comparison of the existing SEIRA-based gas sensors

References	Description	Target	Performance	Remarks
Nishijima *et al.*[13]	Using plasmonic extraordinary transmission	SF_6 only	Detection limitation: 5 ppm; Sensitivity: 0.02% ppm^{-1}	Bulky; require centimeter long optical interaction length;
Nasir *et al.*[14]	Based on a gold/palladium core/shell nanorod metamaterial	H_2 only	Detection limitation: 0.1%; Detection range: 0-40 %;	Visually detectable
Wang *et al.*[15]	Integrating plasmonic nanopatch gold antennas with metal-organic framework	CO_2 only	1800× enhancement factors; Detection limitation: 52 ppm;	Ultrasensitive
Lee *et al.*[16]	Integrating polymer polyethylenimine with photonic crystal slab	CO_2 only	Response time: 2 min; Detection limitation: 20 ppm; Detection range: 0-1000 ppm;	Non-linear output; Easy to reach saturation;
This work	Integrating ZIF-8 with metamaterial absorber	CO_2 & CH_4	Response time: 35 s (CO_2), 60s (CH_4); Detection limitation: 80 ppm (CO_2), 200 ppm (CH_4); Detection range:0-2.5×10^4 ppm	Simultaneous; On-Chip; Multi-gases sensing; high accuracy;

Reference

[1] a) X. Liu, T. Starr, A. F. Starr, W. J. Padilla, *Phys. Rev. Lett.* **2010**, *104*, 207403; b) W. J. Padilla, M. T. Aronsson, C. Highstrete, M. Lee, A. J. Taylor, R. D. Averitt, *Phys. Rev. B* **2007**, *75*, 041102.

[2] S. Y. Ding, J. Yi, J. F. Li, B. Ren, D. Y. Wu, R. Panneerselvam, Z. Q. Tian, *Nat. Rev. Mater.* **2016**, *1*, 1.

[3] a) S. Fan, W. Suh, J. D. Joannopoulos, *J. Optic. Soc. Am. A* **2003**, *20*, 3; b) L. Verslegers, Z. Yu, P. B. Catrysse, S. Fan, *J. Opt. Soc. Am. B* **2010**, *27*, 10.

[4] C. S. Wu, Z. H. Xiong, C. Li, J. M. Zhang, *RSC Adv.* **2015**, *5*, 82127.

[5] G. Khandelwal, A. Chandrasekhar, N. P. M. J. Raj, S. J. Kim, *Adv. Energy Mater.* **2019**, *9*, 1803581.

[6] K. S. Park, Z. Ni, A. P. Cote, J. Y. Choi, R. Huang, F. J. Uribe-Romo, H. K. Chae, M. O'Keeffe, O. M. Yaghi, *Proc. Natl. Acad. Sci. U. S. A.* **2006**, *103*, 10186.

[7] K. E. Dierenfeldt, *J. Chem. Educ.* **1995**, *72*, 281.

[8] Database of the National Institute of Standards and Technology: https://webbook.nist.gov/chemistry/name-ser

[9] D. Liu, D. Zou, H. Zhu, J. Zhang, *Small* **2018**, *14*, e1801454.

[10] L. Zhang, L. Li, E. Hu, L. Yang, K. Shao, L. Yao, K. Jiang, Y. Cui, Y. Yang, B. Li, B. Chen, G. Qian, *Adv. Sci.* **2019**, *7*, 2.

[11] H. Yuan, S. Aljneibi, J. Yuan, Y. Wang, H. Liu, J. Fang, C. Tang, X. Yan, H. Cai, Y. Gu, S. J. Pennycook, J. Tao, D. Zhao, *Adv. Mater.* **2019**, *31*, e1807161.

[12] A. E. Cetin, D. Etezadi, H. Altug, *Adv. Optic. Mater.* **2014**, *2*, 866.

[13] Y. Nishijima, Y. Adachi, L. Rosa, S. Juodkazis, *Optic. Mater. Express* **2013**, *3*, 968.

[14] M. E. Nasir, W. Dickson, G. A. Wurtz, W. P. Wardley, A. V. Zayats, *Adv. Mater.* **2014**, *26*, 3532.

[15] X. Chong, Y. Zhang, E. Li, K.-J. Kim, P. R. Ohodnicki, C. H. Chang, A. X. Wang, *ACS Sens.* **2018**, *3*, 230.

[16] D. Hasan, C. Lee, *Adv. Sci.* **2018**, *5*, 1700581.
